# Multiple lines of evidence suggest the persistence of the Ivory‐billed Woodpecker (*Campephilus principalis*) in Louisiana

**DOI:** 10.1002/ece3.10017

**Published:** 2023-05-18

**Authors:** Steven C. Latta, Mark A. Michaels, Thomas C. Michot, Peggy L. Shrum, Patricia Johnson, Jay Tischendorf, Michael Weeks, John Trochet, Don Scheifler, Bob Ford

**Affiliations:** ^1^ Project Principalis, National Aviary, Allegheny Commons West Pittsburgh Pennsylvania USA; ^2^ Project Principalis Yorktown Heights New York USA; ^3^ Institute for Coastal and Water Research University of Louisiana Lafayette Louisiana USA; ^4^ Project Principalis Travelers Rest South Carolina USA; ^5^ American Ecological Research Institute Manhattan Kansas USA; ^6^ Craighead Institute Bozeman Montana USA; ^7^ Project Principalis Luling Louisiana USA; ^8^ Museum of Wildlife and Fish Biology, Department of Wildlife, Fish and Conservation Biology University of California at Davis Davis California USA; ^9^ Project Principalis Houston Texas USA; ^10^ U.S. Fish and Wildlife Service Falls Church Virginia USA

**Keywords:** audio, bottomland hardwood forests, drones, endangered species, extinction, trail camera

## Abstract

The history of the decline of the Ivory‐billed Woodpecker is long and complex, but the status of the species since 1944, when the last widely accepted sighting in continental North America occurred, is particularly controversial. Reports of Ivory‐billed Woodpeckers have continued, but none has reached the threshold of quality for general acceptance by ornithologists or the birdwatching public. In 2021, the U.S. Fish and Wildlife Service opened for public comment a proposal to declare the species extinct. Here, we present evidence suggesting the presence of the Ivory‐billed Woodpecker at our study site, based on a variety of data collected over a 10‐year search period, 2012–2022. These data are drawn from visual observations, ~70,000 h of recordings by 80–100 acoustic recording units, ~472,550 camera‐hours by as many as 34 trail cameras, and ~1089 h of video drawn from ~3265 drone flights. Using multiple lines of evidence, the data suggest intermittent but repeated presence of multiple individual birds with field marks and behaviors consistent with those of Ivory‐billed Woodpeckers. Data indicate repeated reuse of foraging sites and core habitat. Our findings, and the inferences drawn from them, suggest that not all is lost for the Ivory‐billed Woodpecker, and that it is clearly premature for the species to be declared extinct.

## Graphical abstract

The last widely accepted sighting of the Ivory‐billed Woodpecker in continental North America occurred in 1944. Here, we present evidence suggesting the presence of this species at our study site in Louisiana. Using multiple lines of evidence, including visual observations, acoustic recordings, trail camera photographs, and drone videos, our data suggest intermittent but repeated presence of multiple individual birds with field marks and behaviors consistent with those of Ivory‐billed Woodpeckers.

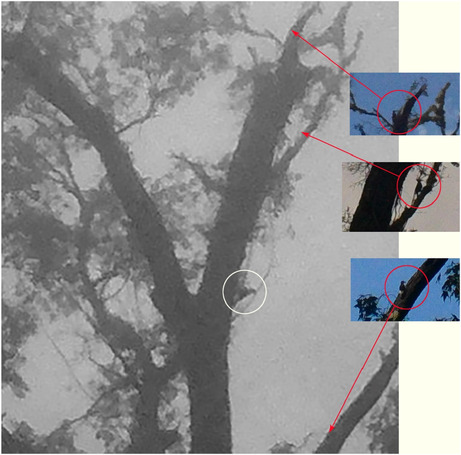

## 1 INTRODUCTION

The history of the decline of the North American population of the Ivory‐billed Woodpecker (*Campephilus principalis*; Ivorybill) is long and complex (Gallagher, 2005; Jackson, 2002; Snyder, 2007). The species historically inhabited mature bottomland forests associated with river basins, and mature upland pine forests, throughout the southeastern United States, with a small, separate population in Cuba currently treated as a subspecies, *C. p. bairdii* (Jackson, 2002) or distinct species (Fleischer et al., 2006). Evidence suggests that the Ivorybill was widespread and perhaps very locally common, moving among ephemeral and widely dispersed areas of optimal habitat with access to recent burns, blowdowns, hurricane destructions, and other areas where the birds foraged, particularly on beetle larvae in dying or recently dead trees (Jackson, 2002).

The Ivorybill was severely impacted by collectors, hunters, and the cutting of bottomland forests and vast expanses of virgin pine forests in the U.S. (Jackson, 2004; Snyder, 2007). By the late 1930s, a documented population count of three territories was known from the Singer Tract, near Tallulah, Louisiana, while a range‐wide search in continental North America resulted in an estimated population of 22 individuals in Florida, South Carolina, and Louisiana (Tanner, 1942), although no additional birds were seen.

The last widely accepted sighting of an Ivory‐billed Woodpecker in North America was in 1944 at the Singer Tract (Hoose, 2004), where Tanner (1940, 1942) had studied the species. Reports of Ivory‐billed Woodpeckers continued, however, with authorities estimating as many as 200 sightings after 1944 (Mendenhall, 2005; USFWS, 2010). Many of these reports were from less well‐known sources, but some were from game wardens, field biologists, and ornithologists. Some observations also included physical evidence, such as photographs, audio recordings, videos, and a feather (Agey & Heinzmann, 1971; Collins, 2017; Lewis, 1988; Lowery, 1974; USFWS, 2010). In 2005, a highly publicized description of seven independent sightings and a video of a possible Ivory‐billed Woodpecker in Arkansas was published (Fitzpatrick et al., 2005). But the identification and the continued existence of the species were strongly debated (Collinson, 2007; Fitzpatrick, Lammertink, Luneau Jr., Gallagher, Harrison, et al., 2006; Fitzpatrick, Lammertink, Luneau Jr., Gallagher, & Rosenberg, 2006; Gotelli et al., 2012; Haney, 2021; Jackson, 2006, 2010; Sibley et al., 2006; Solow et al., 2012). A follow‐up, 2‐year search did not produce additional imagery or documentation widely considered conclusive despite at least 15 reported visual sightings (Fitzpatrick, Lammertink, Luneau Jr., Gallagher, Harrison, et al., 2006; Fitzpatrick, Lammertink, Luneau Jr., Gallagher, & Rosenberg, 2006). Most recently, published evidence suggested that Ivory‐billed Woodpeckers were present in the forests along Florida's Choctawhatchee River (Hill et al., 2006), and a morphometric analysis of a 2010 photo pointed towards an Ivorybill in Louisiana (Luneau, 2021).

None of the published reports and evidence over recent decades resulted in general acceptance that the species persisted anywhere in continental North America (USFWS, 2019), and in 2021, the U.S. Fish and Wildlife Service opened for public comment a proposal to declare the species extinct (USFWS, 2021). Objections to conclusions of the continued existence of the Ivory‐billed Woodpecker among scientists, elements of the birdwatching community, and public media have often focused on two key issues. First, the quality of all reports is so poor that they do not offer decisive proof of a living Ivory‐billed Woodpecker (Hayes & Hayes, 2007; Jackson, 2006; McKelvey et al., 2008; Sibley, 2007). It has been argued that a rare bird needs to be documented with a higher standard of evidence and a greater threshold of physical support than routinely adopted for other species; the USFWS (2021) defined the objective evidence needed to verify the continued existence of the species as “clear photographs, feathers of demonstrated recent origin, specimens, etc.” A second issue in consideration of the persistence of Ivorybills has been the lack of repeatability of observations (Sibley, 2007). The assumption is that if a rare resident species is found, then it should be repeatedly relocated, and that if it is not relocated, then the original observation or record is inadequate to prove persistence.

Here, we draw on 10 years of search effort to address the question of whether Ivory‐billed Woodpeckers might persist in our Louisiana study site. We provide multiple lines of evidence, including visual observations, audio files, trail camera photographs, and drone videos, with evidence suggesting the intermittent but repeated presence of multiple individual birds with field marks and behaviors consistent with those of Ivory‐billed Woodpeckers.

## 2 MATERIALS AND METHODS

Our field research took place in bottomland hardwood forests in Louisiana from 2012 to 2022. Because of the endangered status of the species and ongoing research concerns, we omit specific location details. The search area was defined by mature bottomland forest habitat, previous visual sightings or aural data, and accessibility. The area is a >90 km^2^ mosaic of wooded swamp and bottomlands occupying a system of drainages and backwaters ~10 km in length, and in breadth from 50 m along some of the smaller feeder streams to ~1.5 km in places along the mainstream. This system occurs in a landscape with more remnants of seemingly suitable habitat nearby. The dominant tree species in the semi‐permanently flooded, wooded swamp is bald cypress (*Taxodium distichum*). The dominant tree species in the seasonally flooded bottomland hardwood forest is sweetgum (*Liquidambar styraciflua*). Other common species in the bottomland include several species of red and white oaks, such as cherrybark oak (*Quercus pagoda*), water oak (*Q. nigra*), chestnut oak (*Q. michauxii*), and willow oak (*Q. phellos*), as well as pignut (*Carya glabra*) and bitternut (*C. cordiformis*) hickory, American beech (*Fagus grandifolia*), and American sycamore (*Plantanus occidentalis*). The canopy height rises to ~30 m. Standing and downed dead trees are patchily important components of the landscape. Like almost all bottomland habitats in the southeast, the area has a long history of human use, with most timber extraction having occurred from 1890 to 1940.

Field observations and data reported here were collected through visual encounters, audio detections, the deployment of trail cameras, and the use of drones to record videos. Most fieldwork was concentrated in the October–May period thought to encompass the breeding season of this species (Jackson, 2002).

### 2.1 Visual encounters

Observational techniques that resulted in visual encounters included slowly moving reconnaissance, sitting in place with a view of appropriate habitat, and stakeouts of key areas, points, or cavities where we had seen or heard indications of the possible presence of Ivory‐billed Woodpeckers. No standard protocol was followed for any of these observational techniques, but we were guided by local conditions, and observer experience and availability. Boats were not used due to the number and variety of obstructions in the water, reduced mobility, and inability to also handle recording and other equipment. Field observations focused on the birds occurring in this habitat. Although we carefully noted foraging sign (extensive removal or “scaling” of bark) and potential nesting or roosting cavities, we used these signs to focus our search strategy; we did not quantify or otherwise measure these Ivorybill signs and do not further report on them here.

### 2.2 Audio recordings

From February to April 2019, and December 2019 to April 2020, we deployed AudioMoth acoustic recording units (ARUs; https://www.openacousticdevices.info/audiomoth). Our goal was to use these recordings, machine learning, and open‐source software to identify putative, nasal “*kent*” calls of Ivory‐billed Woodpeckers, and to use the distribution of calls to narrow the search area for locating a nest. Each ARU was placed in a waterproof plastic bag with a desiccant to absorb condensation, and attached to a tree at breast height with a tension strap. ARUs were deployed at ~200‐m intervals across a predetermined grid pattern in the core of our research area and were programmed to operate from before sunrise to 1100, and 1600 to sunset.

In addition, field observers opportunistically recorded possible *kent* calls, as well as “double‐knocks.” Double‐knocks are hard raps or blows, with the second note sounding like an immediate echo of the first (Tanner, 1942); double‐knocks are characteristic of all *Campephilus* species (Jackson, 2002) and have been reported for Ivorybills (Tanner, 1942). Our recordings were made using handheld devices including Zoom H1 and Zoom H4N; the frequency of encounters was not noted.

Audiospectrograms of selected calls and double‐knocks were prepared using Raven Pro software, Version 1.6.4. Results were compared with audiospectrograms prepared with the same software of known Ivory‐billed Woodpeckers recorded by A. Allen and P. Kellogg in the Singer Tract in April 1935 (the “Singer recordings”), and to recordings made by J. Dennis in February 1968 in the Big Thicket of Texas (the “Dennis recordings”). The Dennis recordings are assumed to be of an Ivorybill by the Macaulay Library (Cornell Laboratory of Ornithology), although the bird was not seen while recorded, and some ornithologists differ in their opinions as to the identity of the vocalizing bird.

### 2.3 Trail camera imagery

We used trail cameras in an attempt to capture images of Ivory‐billed Woodpeckers foraging or excavating. Images were obtained using the PlotWatcher Pro Game Surveillance System, Bushnell Trophy Cam Model 119426, Moultrie M8000(i) Digital Game Camera, Stealth Cam DS4K Max, or Stealth Cam DS4K Ultimate. We placed trail cameras strategically at sites where we noted the presence of (a) tight‐barked trees that appeared to have been scaled, (b) trees that were damaged or in poor health and expected to die, or (c) upright or fallen trees of species that are known to be favored for feeding by Ivorybills. Our best results, however, followed placements made when informed by visual or aural encounters with suspected Ivorybills. Cameras trained to capture images of birds foraging in the mid to upper canopy relied on time‐lapse programming at intervals of 5–60 s, while those targeting lower portions of trunks or fallen branches were usually set to a motion‐sensitive setting. Most often, a single trail camera was placed in position to capture activity at a tree, but in some cases, especially where suspected activity had been captured, 2–4 cameras would be placed. This permitted a focus on more sides of the tree, and by programming each camera to different time blocks, we could better avoid taking photos into the sun. Batteries and SD cards were changed as needed or when possible.

No manipulations were made to trail camera images other than adjusting contrast and brightness to the entire image using Photoshop or Apple Photos; there was no attempt to alter the appearance of individual subject birds. GlueMotion software was used to compile still images from trail cameras into time‐lapse videos.

### 2.4 Drone videos

Because we recognized that Ivory‐billed Woodpeckers regularly fly through and over the canopy (Tanner, 1942), and drones have been shown to be effective in detecting putative Ivorybills (Collins, 2018), we hovered a drone in place well above the forest, passively filming the treetops to record birds flying within view of the onboard camera. Hovering the drone at a high altitude, just below the Federal Aviation Administration's maximum height of 122 m (400 ft), minimizes disturbance to birds and other wildlife (Duporge et al., 2021; Weston et al., 2020), and creates a relatively stable platform for the camera that results in less blurring of video images than if the drone were moving.

Selection of flight and video locations was informed by many factors, including available habitat, the configuration of habitat on a landscape scale, accessibility of launch sites, permit requirements, and most critically, our history of aural detections and sightings of putative Ivorybills, and locations of possible foraging signs and cavities. Flights were made primarily near dawn and in the morning hours directly to preselected points where the drone remained in place as long as batteries permitted. Videos were filmed at a shallow (oblique) angle that included treetops up to 800 m away, allowing for a wider field of view and increased opportunity for an encounter with a woodpecker as compared to a directly downward (nadir) view.

Flights during 2019 were made with a DJI Mavic 2 Zoom filming with a 4 K camera, often using the 2× optical zoom lens. In spring of 2020, we began using the Autel Evo II drone with swappable 6 K and 8 K cameras. Due to a smaller sensor, the 8 K camera did not perform well in low light level conditions such as during early morning and on cloudy days, so most videos were recorded with the 6 K camera. Postprocessing of drone videos was minimal; we first cropped the videos using a cropping software (Clideo.com), then we extracted stills using an extraction software (SnapMotion).

## 3 RESULTS

### 3.1 Visual encounters

Skilled, reliable observers associated with our team, all abundantly familiar with Pileated Woodpecker, Red‐headed Woodpecker (*Melanerpes erythrocephalus*), and other birds of the area, reported 16 visual observations deemed by the observer to be probable Ivory‐billed Woodpeckers. Seven of these were of high enough quality that the observer considered the sighting to be definite (See Appendix 1). Although these observations lack photographic verification, many are supported by field drawings. Most observers reported birds in flight with prominent white trailing edges to the wings, or a large bird with a prominent white “saddle” across the lower back (formed by the white trailing edges of the wings when folded across the posterior dorsum) clinging to a tree in the characteristic style of a woodpecker. Nearly every observer noted unique, *brilliant white* plumage, unlike anything seen in any other black and white bird. Most observers had an instant reaction to their sighting, dominated by astonishment at seeing a bird clearly different from any other, and manifested in the realization that in a sharp and focused manner, they needed to record every detail of the experience.

### 3.2 Audio recordings

From February to April 2019, and December 2019 to April 2020, we deployed 80–100 AudioMoth ARUs resulting in ~70,000 h of recordings. The large volume of recordings, and issues encountered in using the “Singer recordings” as a template for machine learning, proved impractical. We were unable to produce distributions of *kent* calls to narrow the search area for locating a nest. Data derived from AudioMoth recordings will be further analyzed and discussed in a future paper.

**FIGURE 1:**
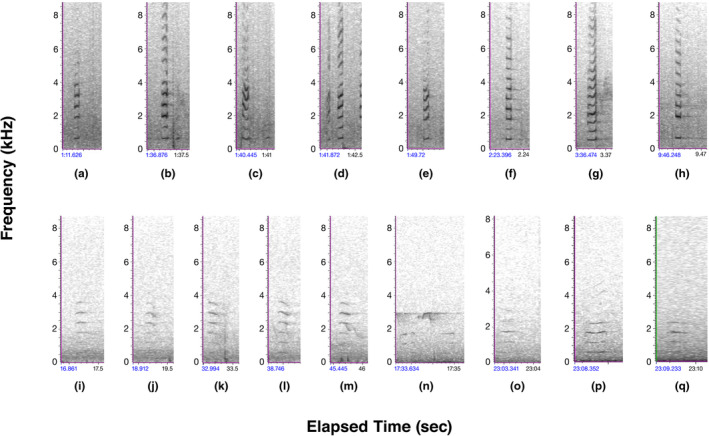
Audiospectrograms of recorded sounds: (a–h) calls extracted from ML6784 recorded by A. Allen and P. Kellogg in the Singer Tract in April 1935 and known to be Ivory‐billed Woodpeckers (the Singer recordings); (i–m) calls extracted from ML104395 recorded by J. Dennis in the Big Thicket, TX, in February 1968 and assumed to be an Ivorybill, although the birds were not seen while recorded (the Dennis recordings); (n–q) calls recorded by M. Courtman with P. Vanbergen in the Louisiana study area in March 2017 using a Zoom H4N handheld recorder. Selections from ML6784 were chosen for the range of *kent* calls given. Selections from ML 104395 were made on the basis of minimal sound signature overlap. Most calls audible on the Courtman recording had at most a tracing with a single frequency at about 1750 Hz. Those selected for the comparison were among the rare tracings with multiple visible harmonics. Audio recordings of the M. Courtman examples are available in Appendix 2.

**FIGURE 2:**
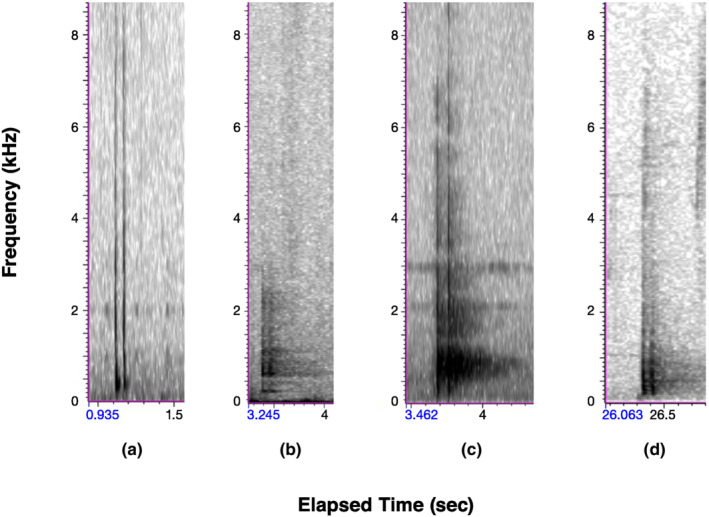
Audiospectrograms for double‐knock drums of (a) putative Ivory‐billed Woodpecker in Louisiana, (b) Pale‐billed Woodpecker (*C. guatemalensis*) in Belize, (c) Powerful Woodpecker (*C. pollens*) in Colombia, and (d) Robust Woodpecker (*C. robustus*) in Argentina. The putative Ivory‐billed Woodpecker double‐knock was recorded in our study area by an AudioMoth ARU on February 18, 2019. The Pale‐billed Woodpecker drum was recorded by P. Driver in Belize in March 2019 (Xeno Canto XC522869); the Powerful Woodpecker drum (Macaulay Library ML90035181) was recorded by D. Uribe‐Restrepo in Colombia in August 2016; the Robust Woodpecker drum (Xeno Canto XC48884) was recorded by B. Lopez‐Lanus in Argentina, date unknown. The audio recording of the putative Ivory‐billed Woodpecker double‐knock can be heard in Appendix 2.

**FIGURE 3:**
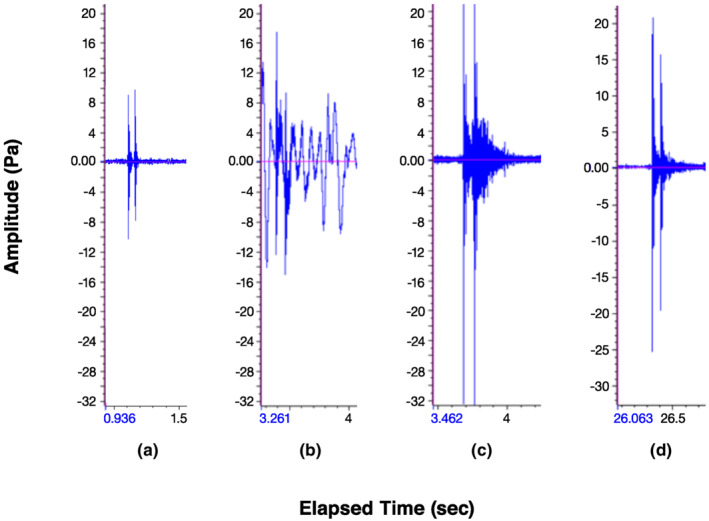
Waveforms for double‐knock drums of (a) putative Ivory‐billed Woodpecker in Louisiana, (b) Pale‐billed Woodpecker in Belize, (c) Powerful Woodpecker in Colombia, and (d) Robust Woodpecker in Argentina. Sources of drums are provided in Figure 2.

Possible *kent* calls and double‐knocks were also heard at infrequent intervals and recorded opportunistically in our study area. We did not quantify the number of each, or score or rank each according to our confidence in identification as putative Ivorybill audio, but possible double‐knocks were heard far more frequently than putative *kent* calls. We present here examples from recordings of a series of *kent*‐like calls and double‐knocks (See Appendix 2) and display audiospectrograms (Figures 1, 2, 3) consistent with Ivorybill reference material obtained from sound libraries. Of particular interest is the unique, very long series of *kent‐*like calls accompanied by double‐knocks recorded with a handheld Zoom H4N recorder in 2017 (the “Courtman recordings”). P. Vanbergen first recorded *kent* calls at this particular location within our study area on March 12. On the morning of March 15, P. Vanbergen and M. Courtman returned to the location and Courtman recorded ~200 *kent* calls and a smaller number of apparent double‐knocks over a 3‐h period (See Appendix 2). Differences in volume among calls made in close temporal proximity indicated that at least two birds were involved. Because the calls emanated from an inaccessible area across a deeply incised waterway, it was not possible to approach the birds. No calls were heard on return visits to the location on March 18 or March 25–28.

All *kent* calls in Figure 1 show a series of equally spaced partials. The nasal quality of the *kent* call arises when most of the sound energy is in the third or higher partial (Pieplow, 2017). In the Courtman recordings from our study site (Figure 1n–q), the third partial is consistently the strongest, and in the 3‐h Courtman recordings (not fully published here), the third partial, at ~1750 Hz, is frequently the only feature visible on the audiospectrogram. In comparison, on the Dennis recordings (Figure 1i–m), the third partial, at about the same frequency as that in the Courtman recordings, is as strong as or slightly weaker than the fourth partial. By contrast, in the Singer recordings (Figure 1a–h), the strongest partial varies from the third to the fifth, and there are frequently 2–4 more or less equally strong partials. The frequency of the third partials on the Singer recordings varies from ~1600 to 2050 Hz. Like the Courtman recordings, the Singer and Dennis recordings share the (mostly) weaker second partial compared with the fundamental.

As a group of three different recordings, the *kent* call audiospectrograms present considerable variability (Figure 1). The Singer recordings (Figure 1a–h) are notable for their short duration *kents*, even though the eight selected calls show flat calls, descending calls, rising calls, and slurred calls. The Courtman calls (Figure 1n–q) have the longest duration *kents*, with the Dennis recordings (Figure 1i–m) having calls of intermediate duration. While most of the Dennis calls are descending, the Courtman recordings show a flat call, a slightly under‐slurred call, and a slightly descending call.

Two characteristics are often mentioned in determining whether a double‐knock can be assigned to an Ivory‐billed Woodpecker: The time interval between the first and second knock is generally 60–120 ms (BWCP, 2019; Hill et al., 2006), and the first knock is generally louder than the second (Jackson, 2002; Tanner, 1942). Looking at the four double‐knock audiospectrograms (Figure 2), we calculated the inter‐knock interval as 61 ms for the presumptive Ivory‐billed Woodpecker (Figure 2a); this is comparable to the inter‐knock interval calculated for the three other *Campephilus* species, including Pale‐billed Woodpecker (72 ms; Figure 2b), Powerful woodpecker (84 ms; Figure 2c), and Robust Woodpecker (59 ms; Figure 2d). We assessed the relative strength of each of the pairs of knocks using their waveforms (Figure 3). The waveforms show that the first knock is slightly louder, as expected for the putative Ivory‐billed Woodpecker in Louisiana (Figure 3a) and Robust Woodpecker (Figure 3d), but for Powerful Woodpecker, the second knock appears louder (Figure 3c).

### 3.3 Trail camera imagery

**FIGURE 4:**
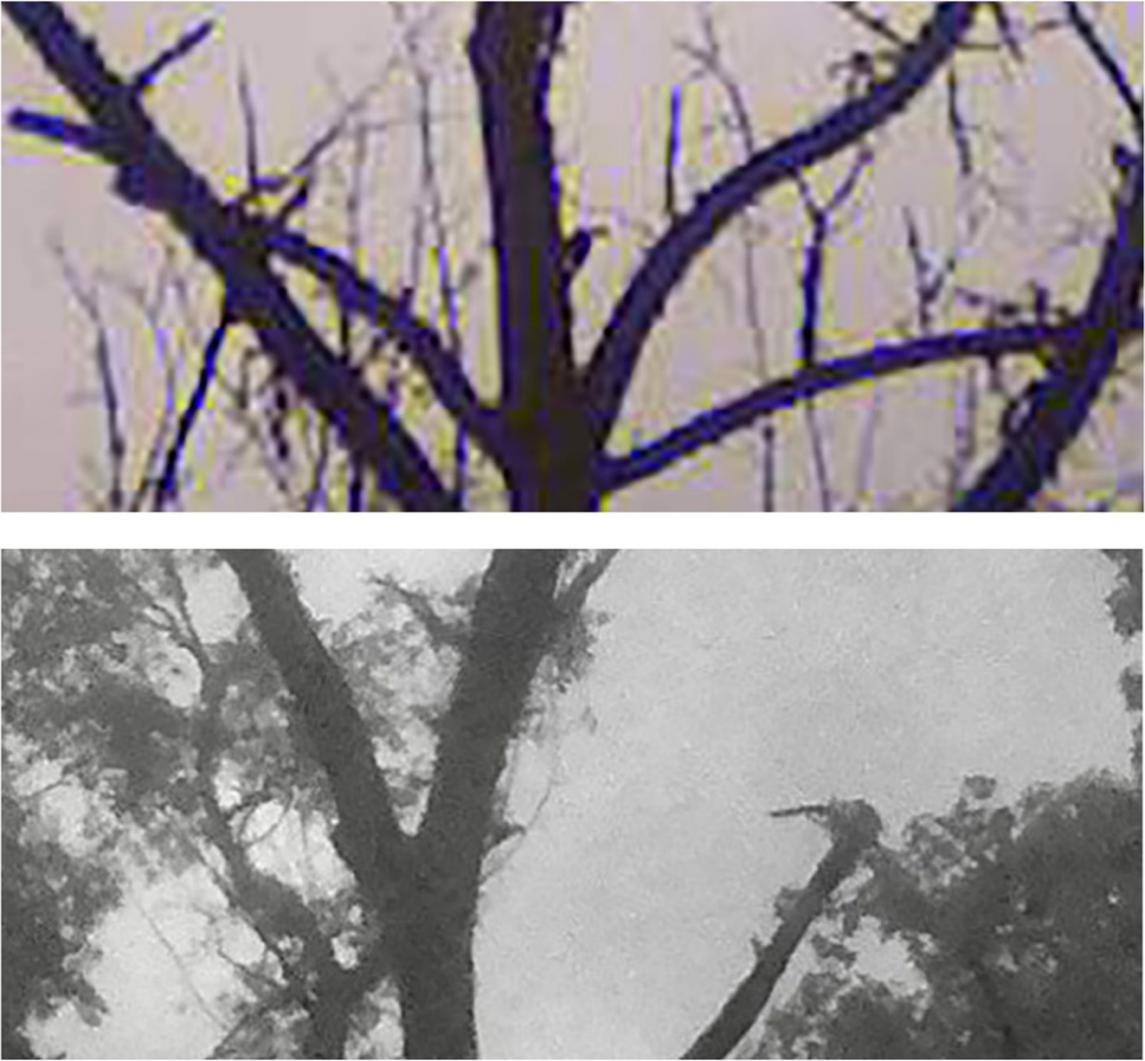
Trail camera photos taken within 50 m of one another on November 30, 2019 (top), and October 1, 2021 (bottom), of apparent Ivory‐billed Woodpeckers showing a prominent white saddle present on the lower part of the folded wings. The image from November 30, taken with a PlotWatcher Pro Game Surveillance System camera, is extracted from the “video” clip composed of trail camera photographs taken at 5‐s intervals and presented in Appendix 3 where a white saddle can be clearly seen in multiple frames. The image from October 1 is selected from a series of images taken by a Stealth Cam DS4K Max showing a pair of birds foraging over a 15‐min period. However, for most of the time, the birds are partially obscured by foliage. Although the white saddle is partially visible in some other frames, this is the only image from the sequence that clearly shows one of the birds in a full, open view.

**FIGURE 5:**
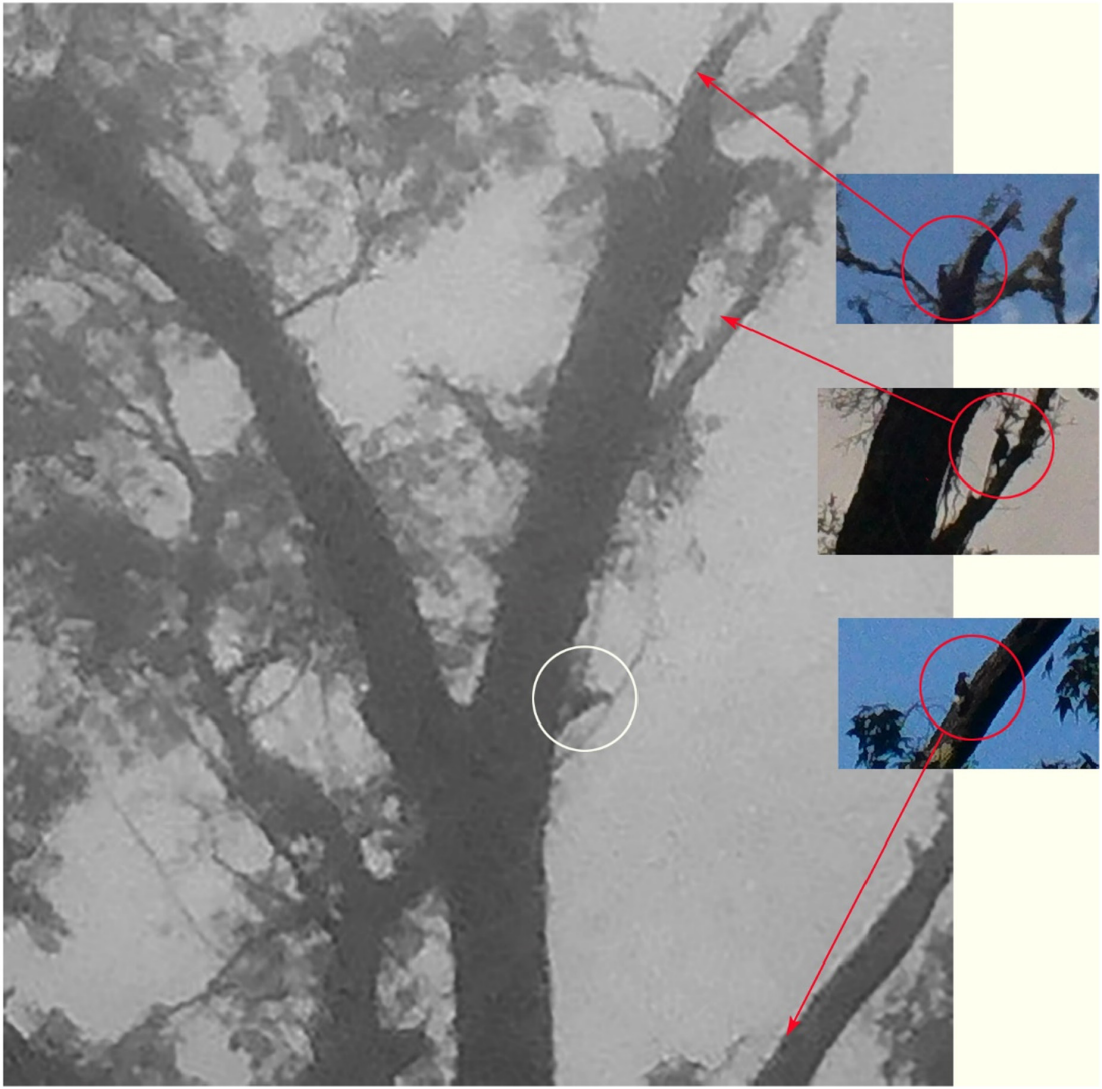
Composite figure comparing the size of three species of woodpeckers to the apparent Ivory‐billed Woodpecker. Inset species were photographed on the same tree, with the same camera in the same place but at different times. These three images were extracted from their original frames and placed as insets on a fourth frame that shows the presumed Ivorybill on October 1, 2021. All woodpeckers here are depicted at the same scale in their original, unedited size. Arrows point to the location of where each bird was located on the tree. Insets include an unidentified small woodpecker (top), a Pileated Woodpecker (middle), and a Red‐headed Woodpecker (bottom). The presumed Ivory‐billed Woodpecker is circled in white without an arrow.

We simultaneously deployed 6–34 trail cameras resulting in ~472,550 camera‐hours of activity. An important series of trail camera photos followed our sighting of an apparent Ivory‐billed Woodpecker landing at ~40 m distance from the observer in a live but declining sweetgum tree on October 27, 2019 (encounter described in Appendix 1). Trail cameras, nearly continuously deployed on this tree since then, subsequently captured photos of possible Ivorybills visiting the tree intermittently from at least November 2019 to February 2020, and then again from September 2021 to December 2021. While many of the images are ambiguous because of distance and light conditions, trail camera photographs taken on November 30, 2019, and October 1, 2021, at this and a nearby tree, both show a bird with a distinct white saddle on the lower back (Figure 4). The white saddle is clearly not “negative space” or skylight shining between the tree and the tail, as the quality of the white is different from that of the sky, and if it were negative space the remaining image of the bird would be an odd and severely truncated body form. Comparative photos of other birds in the same tree taken by the same camera (Figure 5), including an unidentified small woodpecker, a Pileated Woodpecker (*Dryocopus pileatus*), and a Red‐headed Woodpecker, confirm the large size of the putative Ivorybill. The angle of the bird's back to the bole of the tree (~50°) is greater than commonly seen in Pileated Woodpeckers and may reflect the pamprodactyl condition of the Ivorybill (Bock & Miller, 1959; see below), and be characteristic of that species. While the image quality is too poor for precise measurement, the relatively long neck aspect ratio, proposed as characteristic of the Ivorybill (Luneau, 2021), is also highly suggestive, and evident as distinct from Pileated Woodpeckers in many of the photographs taken in the 1930s by Allen and Kellogg (1937) and Tanner (1942).

**FIGURE 6:**
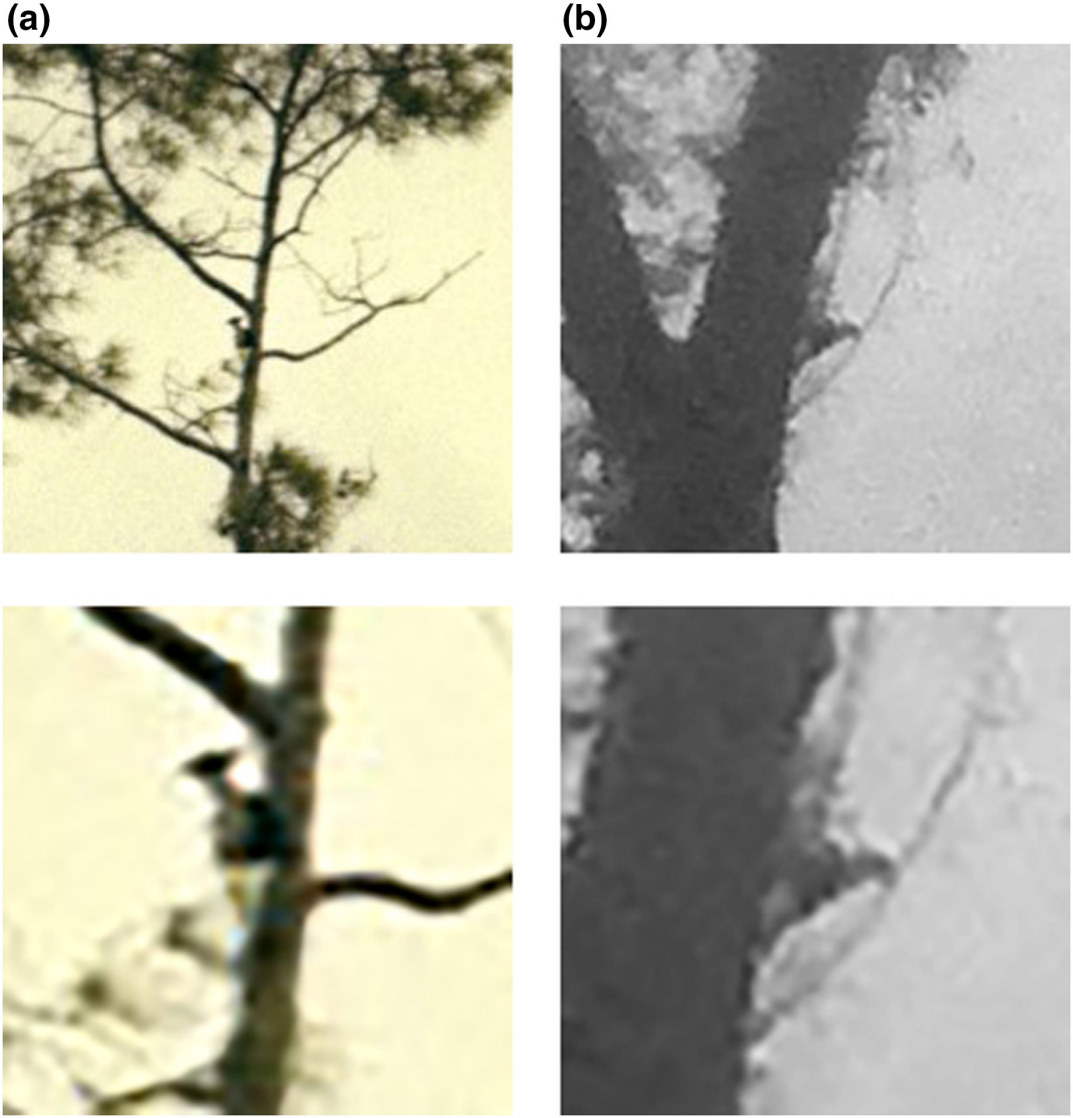
A side‐by‐side comparison of cropped photos from: (a) the unenhanced image of an Ivory‐billed Woodpecker taken by George Lamb in Cuba in 1956 (Gallagher, 2007), and (b) the original, unretouched Project Principalis photo from Louisiana from October 1, 2021. Each photograph is also shown enlarged and further cropped below each original. These comparisons emphasize the similarities of appearance between the known Cuban Ivory‐billed Woodpecker and the presumed Ivorybill from Louisiana where each image was obtained from ground level under challenging field conditions, as opposed to many existing photos of North American Ivorybills that were obtained from cavity‐level blinds (Michaels et al., 2021; Tanner, 1942).

In Figure 6, we compare the putative Ivory‐billed Woodpecker photograph from Figure 5 to one of a known Ivory‐billed Woodpecker from the Cuban population (Gallagher, 2007) that was also photographed at a considerable distance. The remarkable similarities in the images include the angle of the bird to the bole of the tree, the size and shape of the white saddle, and the shape of the crest.

**FIGURE 7:**
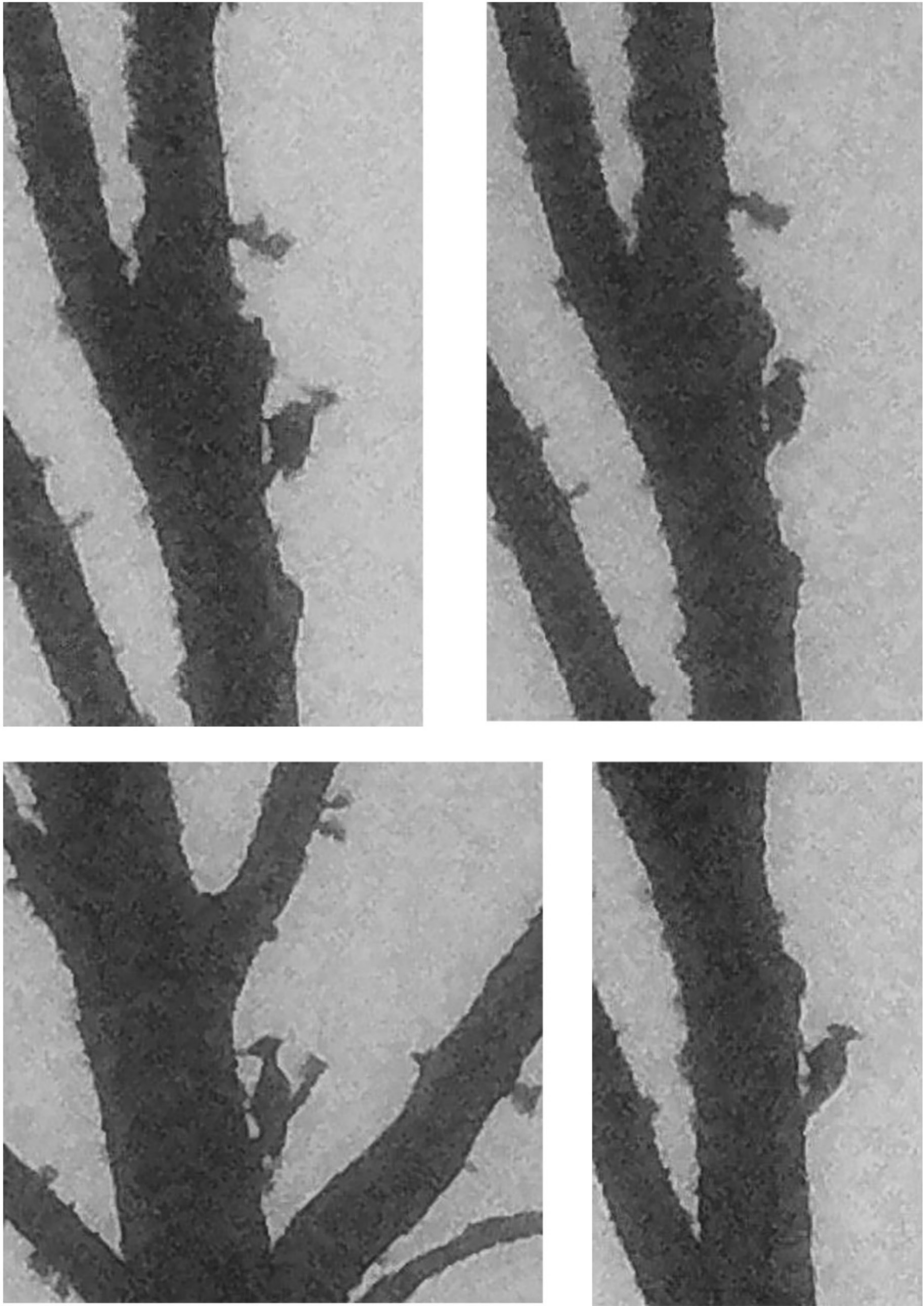
Images taken by a Stealth Cam DS4K on October 14, 2021, of a presumed Ivory‐billed Woodpecker, illustrating the apparent presence of specialized modifications of the feet (pamprodactyly) resulting in a unique position of the legs. The feet are held to the side of the body and are directed diagonally upward and sidewise, with both feet wide apart and forward. Usually, the angle between the tarsi and the horizontal plane is ≤45° and there is an obtuse angle of the intertarsal joint. While a white saddle is not obvious in these early morning, very misty silhouettes, several images suggest its presence.

Camera images obtained on October 14, 2021, show multiple frames with birds exhibiting distinctive traits associated with *Campephilus* woodpeckers (Figure 7). A crested woodpecker with a white saddle, or at least a suggestion of a lighter posterior dorsum, is present in many frames. Most intriguing is that birds in these images appear to have a characteristic body posture resulting from the distinctive morphological adaptations of the feet and legs of *Campephilus* woodpeckers as compared with *Dryocopus* woodpeckers like the Pileated Woodpecker (Bock & Miller, 1959). The Pileated is one of the most unspecialized of the truly arboreal woodpeckers, and when perched on a tree trunk, the legs are positioned more or less beneath the pelvic girdle, the joints are fully flexed, and the tarsi are held well away from the tree trunk.

**FIGURE 8:**
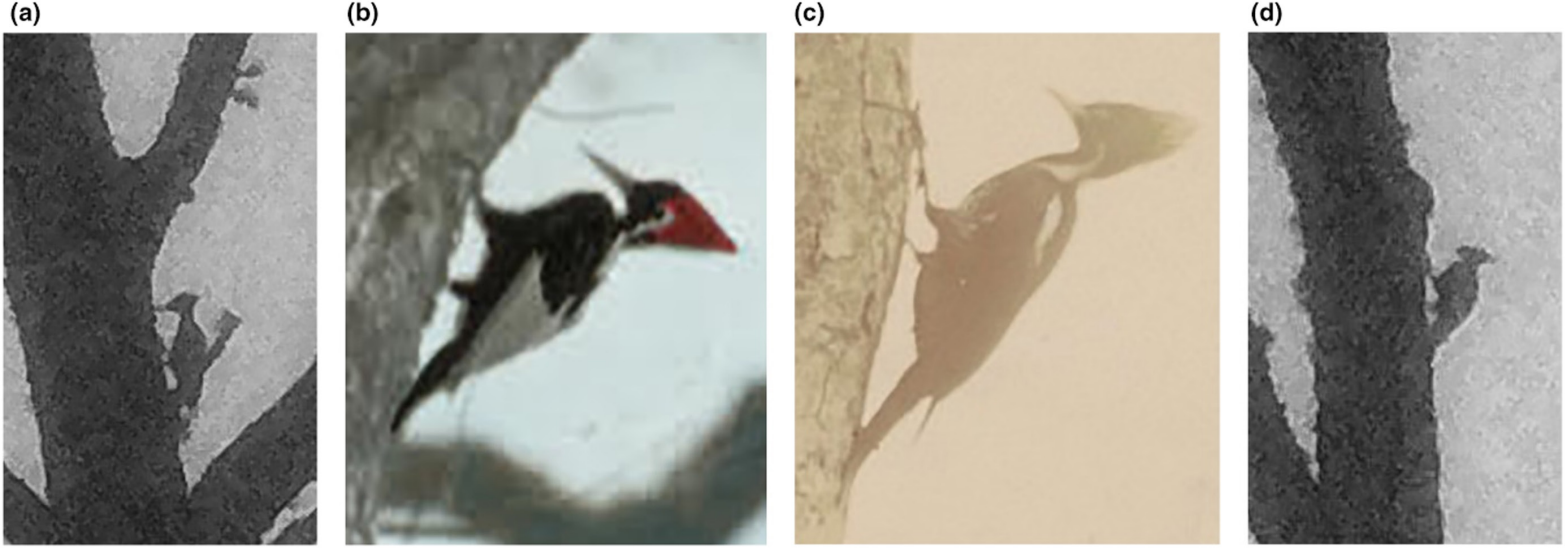
Comparison of photographs taken of apparent Ivory‐billed Woodpeckers in Louisiana from this study (a, d), with a colorized Ivory‐billed Woodpecker, also from Louisiana, but taken by Arthur A. Allen in 1935 (b), and a Pale‐billed Woodpecker (*Campephilus guatemalensis*) taken in Central America (c), also from the Allen Collection. Birds in all photos share the characteristic posture imposed by the unique structure of the *Campephilus* leg and feet. Feet are held to the side of the body and are directed diagonally upward and sidewise, with both feet wide apart and forward. The angle between the tarsi and the horizontal plane is ≤45° and there is an obtuse angle of the intertarsal joint. Photos (b) and (c) are from the James T. Tanner, and the Arthur A. Allen papers, respectively, courtesy Division of Rare and Manuscript Collections, Cornell University Library.

By contrast, the *Campephilus* woodpeckers are characterized by pamprodactyly, a pedal morphology that enables the facultative forward rotation of all four toes (Bock & Miller, 1959). One result of this specialized modification in the structure of the toes of the highly arboreal Ivory‐billed Woodpecker is seen in the position of the legs. The feet and legs are held outward from the body and are directed diagonally upward and sidewise (Figure 8), with both feet wide apart and more anterior relative to the body than seen in other woodpeckers (Bock & Miller, 1959; Tanner, 1940). Usually, the angle between the tarsi and the horizontal plane is ≤45°, and the tarsi often seem to be pressed against the tree trunk. The stance of the Ivorybill generally results then in a more obtuse angle of the intertarsal joint (where the leg bends between the tibiotarsus and the tarsometatarsus) and is evidence of the more efficient scansorial adaptations of the Ivory‐billed Woodpecker compared with the Pileated Woodpecker (Bock & Miller, 1959). This obtuse angle of the intertarsal joint is often visible from a distance and can result in the wider angle of the Ivorybill's back to the bole of the tree than that typically seen in Pileated Woodpeckers. Combined with feet extended diagonally upward and to the side of the body, this stance is readily seen in our comparison of images of known *Campephilus* woodpeckers (Figures 6a and 8b,c), and in our images of putative Ivorybills (Figures 6b and 8a,d).

One of the photo sequences we find most compelling, however, was obtained on November 30, 2019. These trail camera photos involve what appears to be a foraging family group. When viewed in succession (See Appendix 3), the resulting “video” clip appears to show three large, crested woodpeckers moving and foraging together. The “video” is composed of individual trail camera photographs taken automatically every 5 s. Although distance and lighting are difficult, a white saddle can be clearly seen in multiple frames, including a frame extracted and reproduced in Figure 4 (top) showing a woodpecker with a prominent white saddle on the lower part of the folded wings. We note also the proximity of the three birds to one another in the “video,” and their foraging behavior, including movements throughout the tree: on the bole and major branches, and even on smaller branches. Foraging appears to be very active and even acrobatic at times, with birds clinging to the tops, sides, and undersides of the branches. We recorded very similar foraging behavior by at least two birds possessing white saddles on the same tree on October 12, 2021, with very active and acrobatic movements across the tree, including smaller branches (See Appendix 4).

### 3.4 Drone videos

We used drones to document the possible presence of Ivory‐billed Woodpeckers at our study site. We made ~3265 drone flights and recorded ~1089 h of video from July 2019 to December 2022. These videos were taken in areas where we had had recent sightings and had recorded vocalizations suggestive of Ivorybills. On February 23, 2021, a single putative Ivory‐billed Woodpecker was filmed making its way with five short, strong, fast flights through bottomland forest over a ~4‐min period. Three video clips illustrate many important features of this bird. In the first (See Appendix 5), the bird lands, then hops, twists, and turns, along a long, horizontal branch in a manner characteristic of woodpeckers, with a prominent white saddle intermittently seen. Upon reaching the end of the limb, the bird takes off in flight. A very large and well‐defined white saddle is seen at takeoff, followed by multiple frames of the dorsal surfaces of the wings, with black on the leading edge of the wings and white on the trailing edges; the white of the wings is clearly divided by a prominent black body.

In the second flight from February 23, 2021 (See Appendix 6), the same individual bird flies across the lower foreground, then spreads its wings as it prepares to land on an upright tree trunk allowing a full dorsal view of the bird. A cropped and enlarged view of the landing is presented in Appendix 7. Readily visible features include extensive white on the dorsal surface of the wings, black outer primaries visible in some frames, and a clear black body dividing the wings in all frames. At two points in this video, the bird is motionless and perched for ~0.5 s; a clear image is available showing the contrast between the black tail, white saddle, and black torso.

**FIGURE 9:**
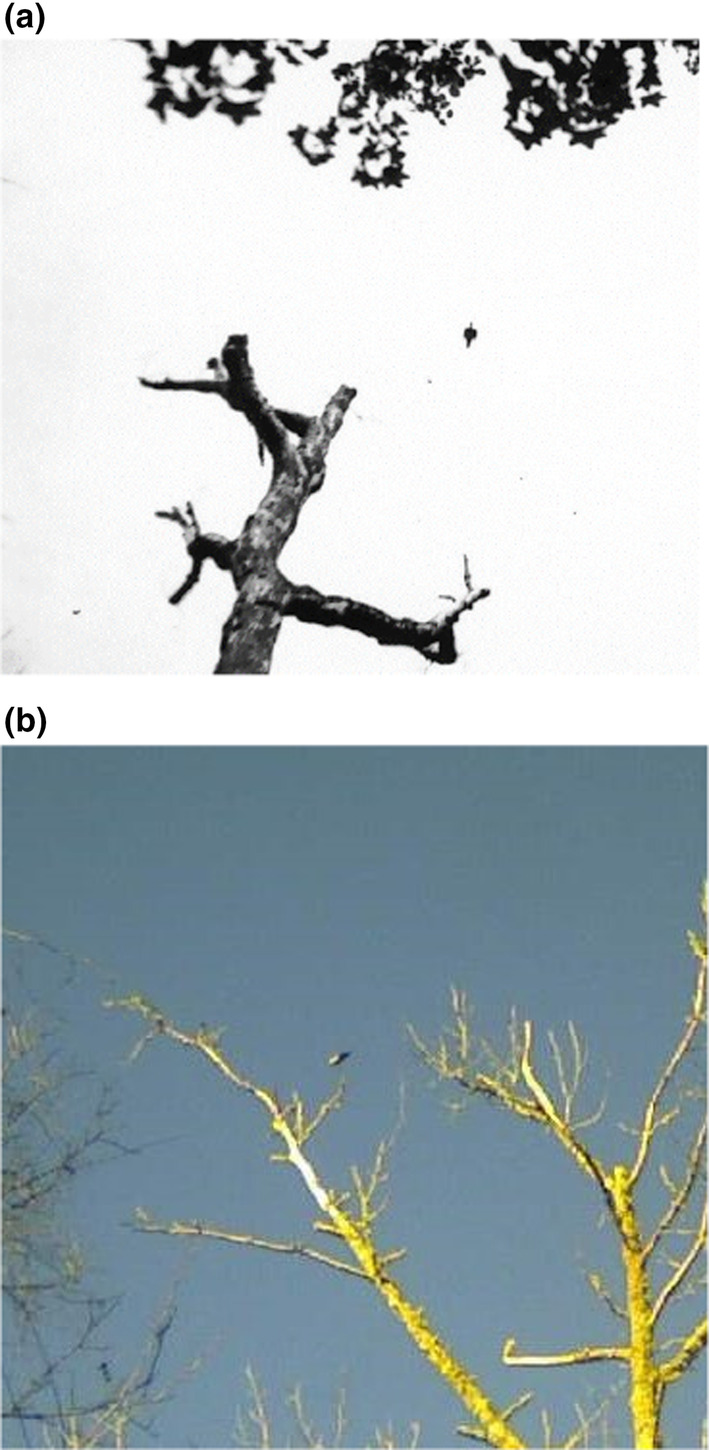
(a) Flight bounding occurs when a bird momentarily stops flapping and temporarily folds its wings onto its back in a tuck position. Flight bounding is not well‐known in Ivory‐billed Woodpeckers, but a photograph by James T. Tanner from April 1939 of an Ivory‐billed Woodpecker demonstrates flight bounding by this species. Photograph courtesy LSU Digital Library, Louisiana, and the Lower Mississippi Valley Collections, Louisiana State University, Baton Rouge, Louisiana. (b) Flight bounding by an apparent Ivory‐billed Woodpecker captured on a PlotWatcher Pro trail camera on December 2, 2019. Although additional confirmatory images from the flight are not available, due to time‐lapse settings on the camera, the image is consistent with what would be expected to be seen when flight bounding. In an Ivorybill, characteristic white plumage appearing along the trailing edge of the wing condenses into a broad, bright white patch across the back of the bird as the bird folds its wings inward across its back. We exclude Red‐headed Woodpecker because the relative extent of white on the folded wings during flight bounding would be reduced because of the Red‐headed's black inner primaries and because the apparent distance to the camera suggests this is a much larger bird. Additional images of flight bounding appear in drone videos in Appendices 6 and 7.

This bird also appears to engage in flight bounding, a behavior most easily seen in Appendix 7, in which the bird stops flapping by temporarily folding its wings onto its back in a tuck position. For a moment, it speeds missile‐like before flapping again. As a bird in sustained flight alternates between flapping and flight bounding, the typical result is an undulating flight path. This is common among woodpeckers, including the Pileated Woodpecker. Flight bounding was documented to occur in Ivorybills by Tanner with a photograph of an adult Ivorybill flying overhead (Figure 9a), and appears in a trail camera photograph of a putative Ivory‐billed Woodpecker from this study (Figure 9b). In Appendices 6 and 7, an extensive white saddle appears on the posterior dorsum when the wings are tucked, a characteristic consistent with an Ivory‐billed Woodpecker and Red‐headed Woodpecker but not a Pileated Woodpecker. The bird then lands on a nearly vertical branch with an upward swoop characteristic of woodpeckers. A bird with a large white patch, bordered above and below by black, can then be seen moving on the branch. It is motionless for ~0.5 s, disappears briefly on the backside of the branch, and then reappears and is again motionless for ~0.5 s when conditions are optimal to see a clear contrast between the black tail, white saddle, and black upper torso. At the very end of the video, as the bird re‐emerges from behind the branch, a white dorsal stripe can be discerned on the black back above the larger white saddle.

To eliminate the possibility that the individual in the video might be a Red‐headed Woodpecker, we calculated the wingspan of the putative Ivorybill (See Appendix 8). The wingspan of a small sample of Ivory‐billed Woodpeckers averages ~78.7 cm (31.0 in; Jackson, 2002), while the wingspan of Red‐headed Woodpeckers averages 41.9 cm (16.5 in; Frei et al., 2020). Our estimate of the wingspan of the putative Ivory‐billed Woodpecker, based on the ratio of the bird's wingspan to the diameter at the breast height of the landing tree, is 74.7 ± 7.9 cm (29.4 ± 3 in).

A very similar set of videos (Appendices 9 and 10) was filmed on October 20, 2022, with the critical difference being that this video includes two birds sharing very similar plumage characteristics consistent with that of Ivory‐billed Woodpeckers. These two birds are clearly interacting, although the video lacks the definition to determine whether they may be a male–female pair or perhaps a parent‐offspring pair.

The large size of the birds in Appendices 9 and 10 is indicated again, this time with the presence of a comparably small Red‐headed Woodpecker just prior to the arrival of the putative Ivorybills. In Appendix 9, the video appears at full speed. At 7.5 s, a Red‐headed Woodpecker flies from the lower‐right to the lower‐middle foreground, briefly lands, and then flies off in the direction from which it arrived. The Red‐headed Woodpecker is identified by its small size, and the dorsal surface of the wings shows a black leading edge with extensive white, but the white is continuous from wing‐to‐wing because of the presence of the prominent white rump. Beginning at 34 s, two putative Ivory‐billed Woodpeckers enter the frame from the mid‐right margin. These two birds, clearly interacting, display field marks consistent with Ivory‐billed Woodpeckers, including the dorsal wing surfaces with a black leading edge, and extensive white trailing edge divided by a prominent black body. A portion of this video is cropped and slowed to three‐quarter speed in Appendix 10.

**FIGURE 10:**
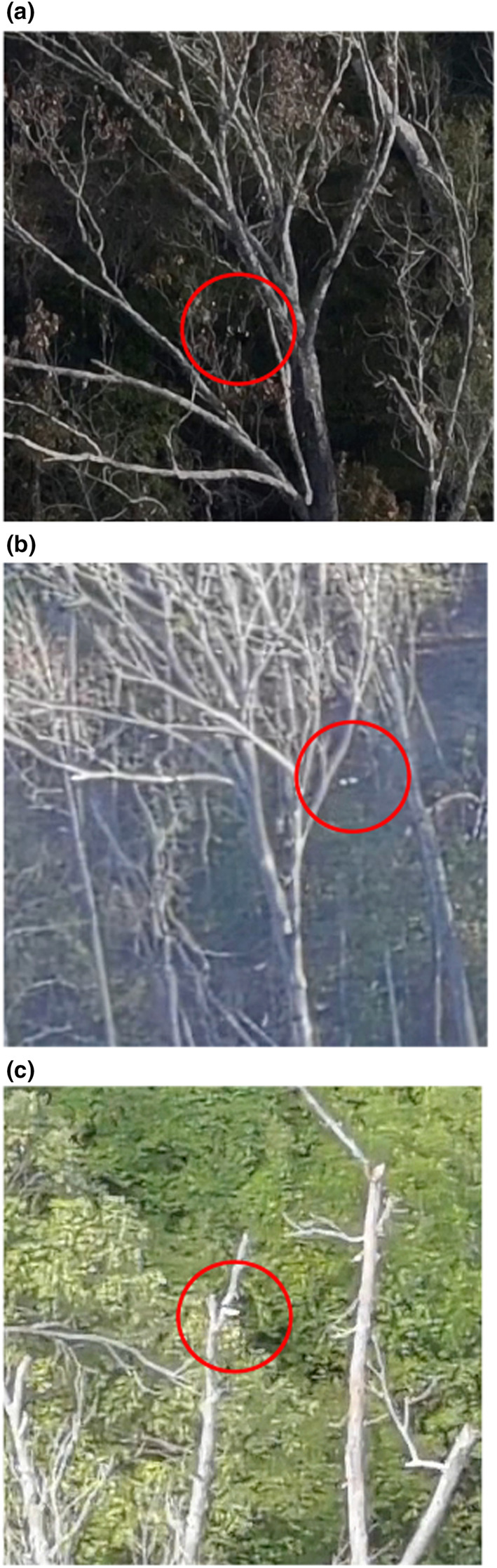
Images extracted from drone video clips, all filmed at our Louisiana study site, depicting landings on upright snags by three woodpeckers: (a) Pileated Woodpecker, (b) putative Ivory‐billed Woodpecker, and (c) Red‐headed Woodpecker. Images have been cropped and sized for comparative purposes but otherwise have not been manipulated. The full video clips of these landings are available in Appendix 7 (putative Ivory‐billed Woodpecker), Appendix 11 (Pileated Woodpecker), and Appendix 12 (Red‐headed Woodpecker). Here, the Pileated Woodpecker (a) displays a very small amount of white on the dorsal surface of the wings. In (b), the dorsal wing surfaces of the putative Ivorybill show extensive white divided by a prominent black body, and a black leading edge to the wing. In (c), the dorsal wing surfaces of the Red‐headed Woodpecker also show a black leading edge to the wing with extensive white, but the white is continuous from wing‐to‐wing because of the presence of the prominent white rump.

Finally, we offer images (Figure 10) and video clips (Appendices 11 and 12) of two species of woodpeckers often suggested as alternatives to possible Ivory‐billed Woodpeckers appearing in camera or video images. These videos show species similar to Ivory‐billed Woodpeckers making swooping landings similar to those made by putative Ivorybills in our videos. These videos were shot using the same drones and in the same habitat as our putative Ivorybills, although natural light conditions may vary. In Appendix 11, a Pileated Woodpecker makes a swooping entry to land on a tree trunk, and in Appendix 12 a Red‐headed Woodpecker leaves a perch on the side of a dead tree, and swoops down and then over to land on an adjacent snag. Readily visible in these videos is the very small amount of white on the dorsal surface of the wings of the Pileated Woodpecker, while in the Red‐headed Woodpecker, the dorsal wing surfaces show a black leading edge with extensive white continuous from wing‐to‐wing because of the presence of the prominent white rump. Stills from these videos are contrasted with a similar still of a putative Ivory‐billed Woodpecker in Figure 10.

## 4 DISCUSSION

Our data, representing diverse lines of inquiry, show multiple images and videos of large, crested woodpeckers. Repeated observations by reliable observers, and suggestive audio, support the possible presence of the Ivory‐billed Woodpecker. The appearance in trail camera photographs and drone videos of woodpeckers with characteristics consistent with those of an Ivory‐billed Woodpecker is also suggestive. Characteristics seen in perched birds include broad white saddles under a variety of lighting conditions, a white dorsal stripe in one instance, and evidence for a unique morphology of the legs and feet resulting in a characteristic stance and body posture. In flight, characteristics include multiple frames of the dorsal surfaces of the wings with black on the leading edge of the wings and white on the trailing edges; white trailing edge of the wings in flight clearly divided by a prominent black body; and large woodpeckers showing a large white patch bordered above and below by black while flight bounding.

Audio recordings of apparent *kent* calls and double‐knocks appear generally consistent with those of known Ivory‐billed Woodpeckers or their congeners (See Appendix 2, Figures 1, 2, 3), but some variability is present. This variability in *kent* calls and their audiospectrograms, in particular, may suggest that one or more sets of recordings are not those of Ivory‐billed Woodpeckers; only the Singer recordings were made with the species in view of the recordists. The Singer recordings, however, present their own limitations as the standard for identifying Ivorybills by call (Hill et al., 2006). These recordings were made with a parabolic microphone that can introduce subtle acoustic distortions (Bruyninckx, 2018). In addition, the recordists were standing near the base of the nesting tree; the birds were agitated and it is reasonable to assume that a bird under extreme stress from the nearby presence of observers may call in unique ways. In fact, Tanner (1942) commented on the variation in calls of Ivory‐billed Woodpeckers, writing that, “The *kent* note, given in a monotone, and slowly or infrequently, is the ordinary call note. When the bird is disturbed, the pitch of the *kent* rises, and it is repeated more rapidly, frequently doubled…” This description of the ordinary note more closely resembles the Courtman and Dennis recordings than the Singer recordings.

In addition, variability among recordings may be a result of differences in habitat or landscapes. Sound propagation from the nest to the recording microphone on the ground nearby, as in the Singer recordings, will be very different from that through the forest, so calls and their spectrograms may appear quite different in unique spaces (Morton, 1975; Wiley & Richards, 1978). More recordings, including experimental manipulations under a variety of conditions, however, would help clarify these and similar questions.

Complementing the audio evidence, the repeated appearance of large woodpeckers in photographs and videos with characteristics consistent with those of an Ivory‐billed Woodpecker is also suggestive. We note that trail cameras, typically designed for close‐range photography, are being used here to take photos at greater distances, and therefore many of our photographs remain ambiguous. For instance, some frames clearly show a white saddle consistent with that expected of an Ivory‐billed Woodpecker, and in some cases, these field marks can be seen in successive or multiple frames. In other cases, however, successive frames may show no white visible for the same birds that showed white in earlier frames. Lighting conditions and position of the bird have been recognized as accounting for the near absence of white in some photos of known Ivory‐billed Woodpeckers (Jackson, 2004), and the angle of the camera to the bird also affects the amount of white appearing in a photograph (Jackson, 2004; Michaels et al., 2021). In this case, we are shooting an apparent Ivory‐billed Woodpecker at a considerable distance, either from a trail camera near ground level or a drone at ~107 m (350 ft), to a bird in the canopy ~20–30 m (65–100 ft) high. Distance, lighting levels, and sun angle can dramatically affect the visibility of the white saddle or any other field mark appearing in photographs or videos in diverse and inconsistent ways.

Large, crested woodpeckers with extensive white plumage consistent with characteristics of an Ivory‐billed Woodpecker are sometimes dismissed as unusual aberrations or leucistic Pileated Woodpeckers, as discussed by Fitzpatrick et al. (2005). However, this argument against the presence of Ivory‐billed Woodpeckers is speculative; no such bird has ever been recorded. Images of leucistic Pileated Woodpeckers do exist, but they all appear to be dramatically different from normal plumage, with extreme amounts of white (or near‐white) plumage, or a mottled appearance. No images exist of a Pileated Woodpecker with a clean white saddle or any other field marks remotely similar to an Ivory‐billed Woodpecker. With the presence of multiple individuals in some videos presented here, the possibility of these putative Ivory‐billed Woodpeckers being leucistic Pileateds is greatly reduced, and the chances of two birds appearing together and showing the same leucistic pattern are vanishingly small.

Related to aberrations are defects or distortions of the video, frequently recognized as “foreign artifacts.” Camera capabilities, including lens quality, camera sensor, and available camera settings for exposure and focus can contribute to video artifacts. Artifacts associated with a “white bleed” and a “black halo” can make it difficult to assess plumage coloration, and the halo effect may be enhanced when cropping distant images as this results in a loss of quality and more pronounced fringing distortions or chromatic aberrations. This may be especially problematic when trying to distinguish the relative position and size of white and black plumage on a flying bird. The flapping motion of the wings, the forward motion of the bird, and ambient or local shading can produce the illusion of greater amounts of white than actually present or a black halo around white patches. This is a particular concern in identifying putative Ivory‐billed Woodpeckers in flight when the amount of white in the wing, and the presence of black on the leading edge and white on the trailing edge of the wing is a critical field mark.

Artifacts associated with camera quality, however, are far less of an issue with recent HD video technology and should be of much less significance in evaluating video shot in 4 K or 6 K HD as we do. In addition, in multiple videos presented here, we see the same plumage patterns, especially when the bird is swooping up to land, or initiating flight bounding when active flapping ceases. In these videos, too, the diagnostic white saddle formed by the white trailing edge of the wing appears *after* the individual has landed and is stationary on the tree, even under various light conditions. This is a strong indication that the apparent white trailing edge of the wing is not an aberration. Finally, as seen in Appendices 11 and 12, our drone videos were also able to capture Pileated (See Appendix 11) and Red‐headed (See Appendix 12) woodpeckers in similar landing flights. In these videos, aberrations are not an issue, the distribution and extent of black and white plumage is as might be expected for these species and is very unlike that of the presumed Ivory‐billed Woodpeckers.

The variety of evidence we have gathered over many years indicates repeated re‐use of foraging sites and core habitats and offers unusual repeatability of detections of putative Ivory‐billed Woodpeckers. The lack of repeatability of observations has been raised in the past to dismiss purported Ivorybill sightings. For example, countering claims around the Luneau video from Arkansas, critics suggested that, “experience with other rare birds, especially resident species, suggests that any valid sighting should very quickly lead to more sightings” (Sibley, 2007). This criticism was lodged, despite the fact that the Luneau video followed a series of sightings and was itself followed by additional sightings and acoustic recordings (Fitzpatrick et al., 2005). Repeatability in our observations is seen at a variety of scales. All of the observations reported here took place in a single forested block and a single watershed. Almost all of the encounters reported here occurred within 1.6 km of one another; the majority of the best trail camera photos were taken over two, 3‐month periods on the same tree; and drone videos were taken over a several‐year period.

Our trail camera “videos” and drone videos of evidently socially bonded and very active foraging by two and three large, crested woodpeckers are extraordinary and distinctly atypical of Pileated Woodpecker behavior. This intraspecific behavior may support the identification of these birds as possible Ivory‐billed Woodpeckers. Ivorybills reportedly show no indication of being strongly territorial (Tanner, 1942). In the Singer Tract, home ranges did not appear to be defended during the breeding season, and wandering birds that were encountered seemed to be tolerated by resident birds. In addition, Sonny Boy, the male offspring that Tanner banded in 1937, remained with his family group for a full 2 years after fledging (Michaels et al., 2021). By contrast, the Pileated Woodpecker generally appears to be territorial year‐round, only tolerating birds from other territories at distances of >100 m (Bull & Jackson, 1995). Adult Pileateds typically drive young away from the territory in the fall, often as early as September, but anecdotal reports do exist of three Pileateds together during winter months (Bull & Jackson, 1995). Our observations of three birds appearing just a few meters apart (See Appendix 3), well after a presumed fledging period and for an extended time, is more consistent with an Ivorybill family group than an unusual Pileated or mixed‐species group but should not be considered definitive. However, considering that we see white on the wings of birds in successive frames (Figure 4), even at a considerable distance and under poor lighting conditions, is consistent with these sequences including Ivory‐billed Woodpeckers.

In addition to the evidence of a family group, the observed foraging behavior is distinctly unlike that of a Pileated Woodpecker. Pileateds select large‐diameter trees (Bull & Jackson, 1995; Newell et al., 2009), and dead trees are used out of proportion to availability (Newell et al., 2009). Large rectangular excavations are characteristic; these can be >30 cm in length (Bull & Jackson, 1995). Although Pileateds may also glean and peck, their bark scaling behavior is a distinctly uncommon activity in Louisiana bottomlands (Newell et al., 2009). Pileated Woodpecker foraging tactics are rather slow and methodical, and concentrated on the bole and major branches of large trees, as the species avoids trees in smaller size classes (Newell et al., 2009). The foraging style of the Ivory‐billed Woodpecker seems to be largely undescribed, other than the importance of scaling of the bark of hardwoods (Tanner, 1942). It is unclear from the literature whether foraging as active as we document is typical of Ivorybills, but our subsequent careful inspection of the smaller branches of the tree where the putative Ivorybills were photographed did reveal extensive scaling of even the smaller branches in the canopy. Furthermore, photographs taken by Tanner in 1939 similarly reveal a group of three Ivorybills foraging together on a tree at the same time, while also documenting that the three birds were also less than 1 m apart from one another (Michaels et al., 2021).

Such active foraging behavior would be enabled in the Ivory‐billed Woodpecker by the unique foot function resulting from the pamprodactyl condition allowing the acrobatic hanging while foraging. This might be the case because when the bird is climbing on smaller limbs, the feet can encircle the limb and thus obtain better support (Bock & Miller, 1959); however, functional studies and comparative videos are lacking. The ability to rotate the toes forward, and the angle and lateral direction of the tarsi, contrasts markedly with tarsus positioning in Pileateds, and may be extremely important in terms of distinguishing these two species behaviorally and morphologically. The legs and feet of Ivory‐billed Woodpeckers are enormous compared with those of Pileated Woodpecker, with a unique angle and direction of their placement in a perched bird, an underappreciated fact that all photos of Ivorybills bear out (Bock & Miller, 1959). In part, this may relate to the physics of vertical perching and climbing while holding up the much larger body mass of Ivorybills compared with Pileateds; the few examples of Ivorybill mass suggest that they are ~60% heavier than Pileateds, a scale consistent with the 15% difference in linear measurements of the two species. Underscoring this point is the striking similarity between the posture of the known Ivory‐billed Woodpecker in Figure 6a and that of the putative Ivorybills shown in Figures 6b and 7. These data suggest that the posture of a perched woodpecker may be a useful identification clue in situations where lighting or distance makes it hard to observe plumage details with clarity (Artuso, 2016).

In addition to foraging behavior, flight characteristics may also be used to aid the identification of these birds. High speed and direct flight were previously noted in the Ivory‐billed Woodpecker (Allen & Kellogg, 1937; Tanner, 1942), and may be similar to that seen in the putative Ivorybill in Appendices 5 and 6. By contrast, Pileated Woodpecker flight is characterized as “rather slow, but vigorous and direct” (Bull & Jackson, 1995). Flight bounding is also known from the Pileated Woodpecker but is not mentioned in the historical literature of the Ivory‐billed Woodpecker (Collins, 2011). A 1956 video of the closely related Imperial Woodpecker (*C. imperialis*), however, shows flight bounding in that closely related species (Lammertink et al., 2011), and a 1939 photograph by Tanner of an adult Ivorybill flying overhead (Figure 9a) is evidence that there are moments when the wings are folded on top of the body. We provide drone videos illustrating apparent flight bounding in a putative Ivory‐billed Woodpecker (Appendices 6 and 7), and a trail camera photograph showing an apparent dark bird with a pronounced white saddle formed by folded wings in flight (Figure 9b) may also refer to an Ivorybill.

We suggest that our observations help explain the twin problems of why the Ivory‐billed Woodpecker has been so difficult to detect and to relocate or re‐encounter over the past 80 years. Assuming that this species does still exist, it is obviously extraordinarily rare. Historical reports suggest that the Ivory‐billed Woodpecker was always scarce (Jackson, 2002), and famously vagile and unpredictable. It was known to colonize or utilize rich but ephemeral resources associated with recent burns, hurricane blowdowns, and floodwaters where dying or recently dead trees hosted favored beetle larvae (Jackson, 2002). This likely helps to explain the unusual mobility of the species that have contributed to the difficulty in locating and re‐encountering the species. This may explain, too, the apparent ~2‐year gap in foraging on one of our nearly continuously monitored trees, supporting the reported intermittency in woodpecker movements, and likely, the phenology of prey. Continued, long‐term monitoring of trees utilized by putative Ivorybills is warranted to better understand woodpecker movement and foraging patterns.

Difficulties in detecting and relocating putative Ivorybills hinge, however, on the misperception that, if present, the Ivorybill is relatively easy to find—a misperception that extends at least as far back as Tanner (Tanner, 1942). Tanner was a meticulous observer, but he apparently never located an Ivory‐billed Woodpecker outside the Singer Tract, despite his numerous searches throughout the southeast (Bales, 2010; Tanner, 1942). Tanner (1942) noted that “the difficulty of finding the birds, even when their whereabouts was known … limited the number of observations.” Nonetheless, the misperception emerged, sometimes fueled by Tanner himself, that the Ivorybill was noisy and easy to find. However, this view was largely based on a single noisy family group that was annoyed with the human intruders below their nest and therefore easily recorded by Tanner and Allen (Tanner, 2001).

Misperceptions on the ease of finding the Ivorybill extend to the frequent argument that, in the modern era, it is unlikely that a large, distinctive woodpecker could escape the sights, cameras, and recorders of birdwatchers and other people who recreate or work outdoors in remote areas (Kaufman, 2020; Roberts et al., 2010; Sykes, 2016). Even with the popularity of birdwatching, however, birdwatchers are not everywhere (LaSorte & Somveille, 2020). The eBird citizen science program (https://ebird.org/home) has amassed >44 million checklists (eBird, 2021). While the most thorough coverage occurs in North America, modeling of the range and relative abundance of individual species at a 3 km spatial resolution results in areas of “no predictions” because there are an insufficient number of qualifying checklists to assess whether a species is present or absent (eBird, 2021). While eBird checklists occur at easily accessible places in the vicinity of our study area, no eBird checklists occur from within our specific area.

Beyond the questions of detection and documentation, our data offer insights into how the ecology and behavior of surviving Ivory‐billed Woodpeckers might contribute to the difficulty in finding or re‐finding this species. We know that, if present, the Ivorybill would inhabit some of the most difficult to access habitats in the United States, and that mature bottomland forests would be a core component of that habitat. Our observations of putative Ivorybills show high speed and direct flights, and long intermittency in detections. These behaviors are suggestive of a species with a vast home range and of individuals that are accustomed to utilizing dispersed and likely fragmented habitats. Home ranges may vary seasonally, but the Ivorybill pair studied in the Singer Tract may have had a range up to four miles or more in diameter (Tanner, 1942). Ivorybills have also been reported to wander over even greater distances and to cross cutover and otherwise unsuitable habitat (Lamb, 1957; Tanner, 1942). Data presented here of putative Ivory‐billed Woodpeckers support evidence that the species moves widely among dispersed areas of optimal habitat with ephemeral resources occurring in dying or recently dead trees (Jackson, 2002).

## 5 CONCLUSION

We conclude that multiple lines of compelling evidence suggest that Ivory‐billed Woodpeckers persist in our Louisiana study site. Cumulatively, our visual observations, audio files, trail camera photographs, and drone videos, suggest the intermittent but repeated presence of multiple individual birds with field marks and behaviors consistent with those of Ivory‐billed Woodpeckers.

The habitat conditions described above apply to many places in the American Southeast (USFWS, 2010). If the Ivory‐billed Woodpecker continues to survive in Louisiana, this has conservation management implications not only in that state but also widely within the historic range of the species. We expect that Ivorybills persist in some of these other places also, if not permanently then episodically. Their numbers cannot be expected to improve unless many more large and continuous bottomland hardwood forests are actively or passively managed to exhibit old growth characteristics. Forested tracts must be large enough and numerous enough that ecological changes caused by natural catastrophic events, such as fires (Bedel et al., 2013), and floods or hurricanes (Doyle et al., 1995; Faulkner et al., 2007), will allow surviving Ivory‐billed Woodpeckers opportunities for a diversity of habitats, including mature bottomland hardwoods. The quantity and distribution of habitat must also take into account changes wrought by anthropogenic climate change and its effects on hydrology, moisture and drying cycles, and severe storm events. Only then, can there be an expectation of a larger number of populations or subpopulations of this iconic species.

The report contained here is not the end of our efforts. We are encouraged and energized by what we have discovered and accomplished. We are optimistic that technologies will continue to improve our outcomes, including documentation through environmental DNA and other physical evidence. We believe that our intentional and systematic survey design is paying off through complementary lines of investigation. Our findings begin to tell a larger story not just of whether the Ivory‐billed Woodpecker persists in Louisiana, but how it has survived and why its survival has been so difficult to document. Finally, we also believe that our methodologies can be translated to other sites, thus offering opportunities for additional documentation of the species. Our findings, and the inferences drawn from them, suggest that all is not lost for the Ivory‐billed Woodpecker and that it is clearly premature for the species to be declared extinct.

## AUTHOR CONTRIBUTIONS

**Steven C. Latta:** Conceptualization (lead); data curation (supporting); formal analysis (supporting); funding acquisition (lead); investigation (lead); methodology (lead); supervision (equal); writing – original draft (lead); writing – review and editing (lead). **Mark A. Michaels:** Conceptualization (lead); data curation (lead); formal analysis (lead); funding acquisition (supporting); investigation (lead); methodology (lead); supervision (equal); writing – original draft (supporting); writing – review and editing (supporting). **Thomas C. Michot:** Data curation (supporting); formal analysis (supporting); investigation (supporting); methodology (supporting); writing – review and editing (supporting). **Peggy L. Shrum:** Data curation (supporting); formal analysis (supporting); investigation (supporting); methodology (supporting); writing – review and editing (supporting). **Patricia Johnson:** Data curation (supporting); formal analysis (supporting); investigation (supporting); methodology (supporting); writing – review and editing (supporting). **Jay Tischendorf:** Data curation (supporting); formal analysis (supporting); investigation (supporting); methodology (supporting); writing – review and editing (supporting). **Michael Weeks:** Data curation (supporting); formal analysis (supporting); investigation (supporting); methodology (supporting); writing – review and editing (supporting). **John Trochet:** Data curation (supporting); formal analysis (supporting); investigation (supporting); methodology (supporting); writing – review and editing (supporting). **Don Scheifler:** Data curation (supporting); formal analysis (supporting); investigation (supporting); methodology (supporting); writing – review and editing (supporting). **Bob Ford:** Data curation (supporting); formal analysis (supporting); investigation (supporting); methodology (supporting); writing – review and editing (supporting).

## CONFLICT OF INTEREST STATEMENT

The authors declare no conflicts of interest.

## Figures and Tables

**FIGURE A1:**
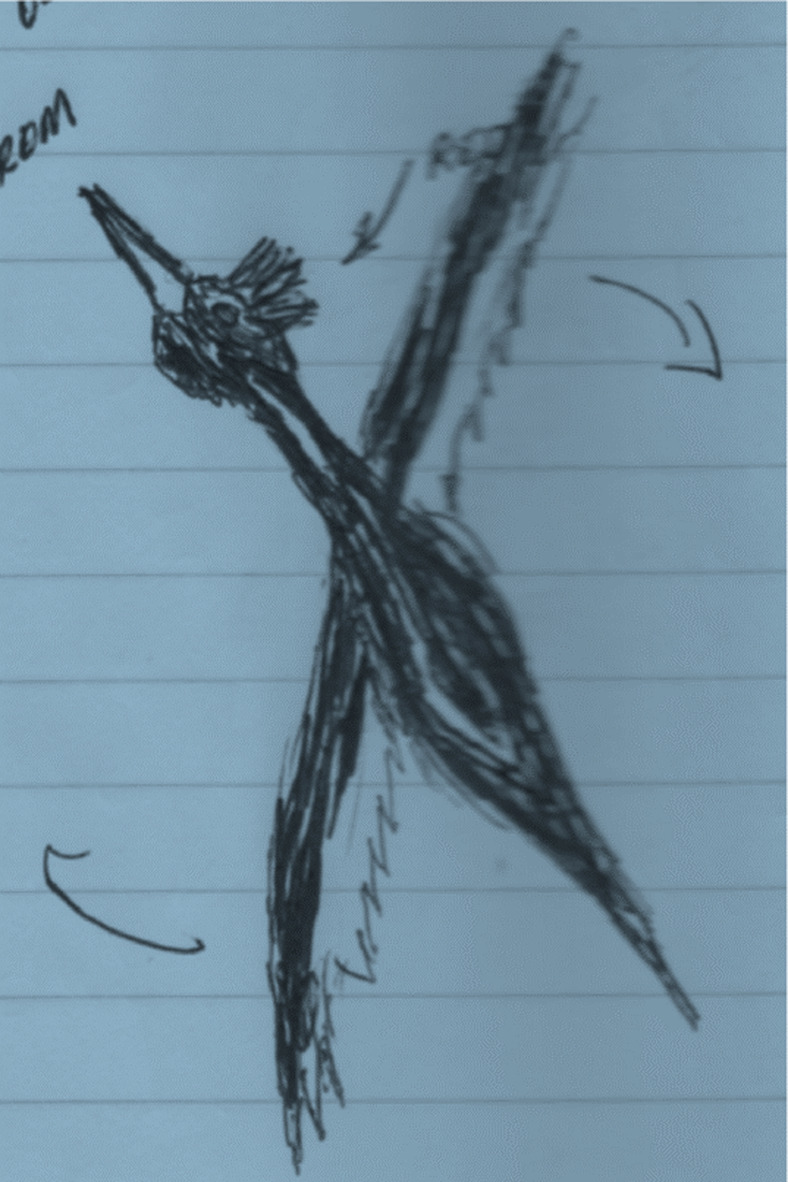
Sketch made by Frank Wiley on April 3, 2015, based on an observation of a bird flushed from a fallen log lying in the water or possibly from along the water's edge.

**FIGURE B1:**
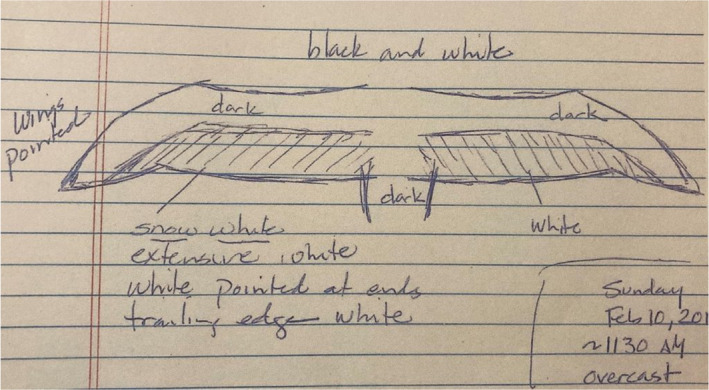
Field drawing by S. C. Latta of the bird he encountered on February 10, 2019.

**FIGURE C1:**
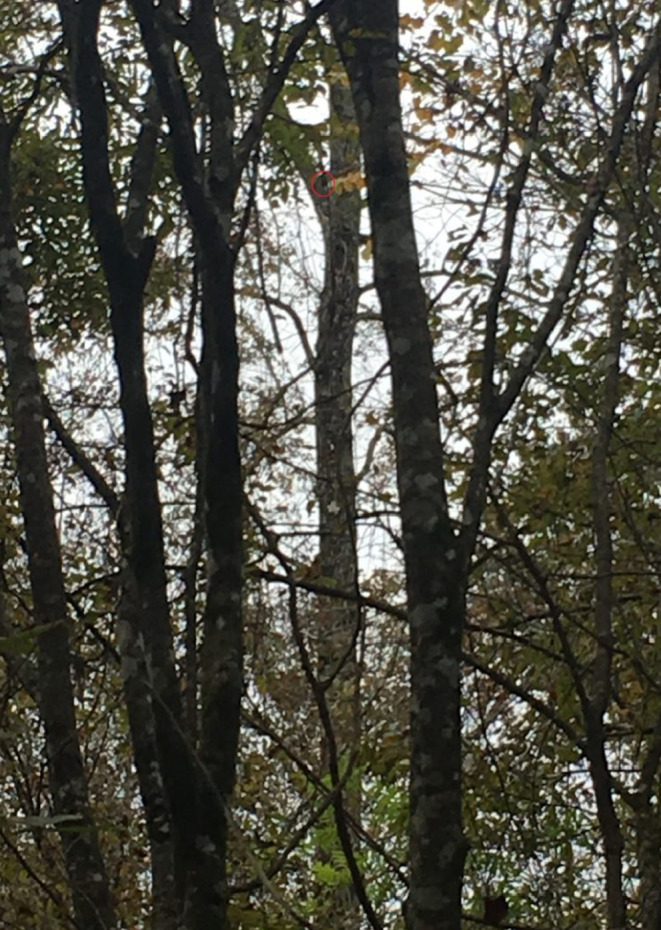
Cell phone capture of possible Ivory‐billed Woodpecker at upper center (circled in red), obtained in conjunction with sighting on October 27, 2019.

**FIGURE D1:**
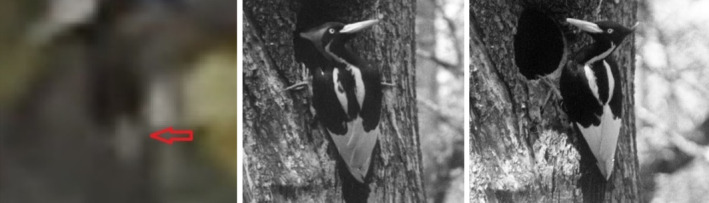
At left, a cropped and enlarged version of the same photograph appearing in Figure C1. The red arrow points to the white saddle on the bird's lower back, with a shape very similar to those in historical Ivorybill photographs from 1935 of a male (center) and female (right). The central portion of the white saddle extends anteriorly leading toward faintly visible dorsal stripes in the photograph at left, similar to the historical photographs and significantly different from those of Red‐headed Woodpeckers in which the central portion of the white saddle does not extend anteriorly. Historical photographs (male: ML72932681; female: ML72932701) by Arthur A. Allen, used by permission of Macaulay Library at the Cornell Lab of Ornithology

**FIGURE E1:**
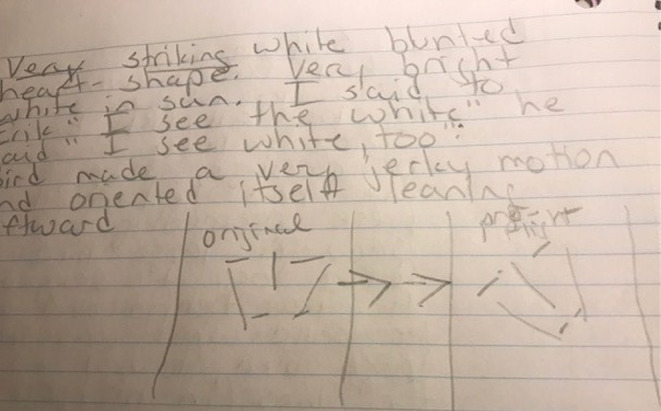
Field notes from Peggy Shrum's observation on February 8, 2020.

## Data Availability

The authors confirm that all of the data supporting the findings of this study are available within the article and its appendices.

## APPENDIX 1

### Visual encounters

The following are first‐hand reports of apparent Ivory‐billed Woodpeckers in our search area. Our team reported 16 visual observations of probable Ivory‐billed Woodpeckers, seven of which were of high enough quality that the observer considered the sighting to be definite. Those seven are described here. One (by Frank Wiley) occurred in 2015, before consistent fieldwork was initiated, but it is included here for its quality and for completeness.

#### From Frank Wiley, co‐founder of Project Coyote (now deceased; as re‐told by Mark Michaels)

On April 3, 2015, Frank and I hiked into the area we called the “hot zone.” We did some playbacks of Ivorybill *kent* calls at approximately 0800, although I did not record anything about them in my notes. We then proceeded walking in a more or less southerly direction with Frank in the lead by about 10 yards; I was walking slowly and looking up and to my right. As we approached a body of water, Frank stopped suddenly and blurted something unintelligible. I caught up with him, and he said he had gotten a very good look at a male Ivory‐billed Woodpecker that had flushed, presumably from a fallen log lying in the water or possibly from along the water's edge. The distance to the log was no more than 20 yards. I handed Frank my field book so that he could draw what he saw and record his observations (Figure A1).

In addition to the sketch, I present a transcription of Frank's description; I made a few redactions related only to the specific location of the sighting. These notes were made immediately after the sighting and without reference to a field guide. Frank immediately noted:

1. A big, traffic cone‐shaped *WHITE* bill (3”ish?)
2. Solid black head/face—light colored eye (whitish)
3. Bright red, crimson crest—puffed up (not what you'd expect)
4. Stripe on face beginning below/behind the eye
5. Stripes on back form chevron over rump
6. Wings long/thin; shallow, rapid flaps
7. Rear 1/3 to 1/2 of wings white, all the way out to primaries
8. Long, tapered tail

Not included in the description was his estimate that the sighting lasted 2–3 s; the bird, was flying in the open for perhaps 9 m (10 yards) as it flew upward, crossing the water, and then into an opening in the woods.

Later that same day in an email exchange, Frank added the following comments: “At the first sign of movement, I assumed a Wood Duck had flushed, looked in that direction, and immediately saw the crimson red of the crest. I then thought, PIWO (Pileated Woodpecker) but noticed the big white traffic cone bill, and an almost entirely black face. There was a white stripe that started below/behind the light (I got the impression of white—not yellow) colored eye. The crest was not ‘groomed’ as is usually seen in most of the artwork—rather it was puffed up as if the bird were agitated.”

#### From Steven Latta

On February 10, 2019, I paired up with Mark Michaels to deploy automated recording units on a predetermined grid in prime, bottomland forest habitat. This was the heart of the project's “hot zone” where fleeting glimpses and enticing *kents* had been reported. At about 1130, we had stopped momentarily when I happened to turn my head perhaps a quarter turn and caught sight of a large bird flying off about 75 yards distant. I quickly completed my turn, squared up, and froze. I did not even think to pick up my binoculars that were around my neck. Based on field notes, the bird was seen in the lower midstory, flying away from me, but angling up as though it had launched from lower down. The bird flew straight and strong with steady but unhurried flaps, but always angled up, so I always had a full view of the bird from wingtip to wingtip and across the entire dorsal area. Much to my advantage, too, the bird flew through a clear corridor or gap in the forest, so for much of its flight my view was not obscured by trees or branches. At the end of this flight, which I estimated lasted perhaps 5–6 or 8 s, the bird appeared to “pull up” in classic woodpecker fashion as it approached the canopy of a pair of very large hardwood trees. My impression was that the bird was pulling up to land on the trunk of one of these trees, but I could not see the landing because of the intervening canopy.

The wings of the bird I saw were very long and relatively narrow, unlike a raptor or a Great Blue Heron that was seen nearby. I was very impressed by the very large size of the bird, the long‐pointed wings with a slight bend at the end of the wing, and the strong, direct flight. More than anything though, I was struck by the black and white pattern, and the brilliant snow white of the wings. The white formed a heavy bar across the entire trailing edge of the wing, with the white forming a point at the end of the wing where it met the black of the forewing.

I did not notice the head of the bird, perhaps because the bird was flying away from me, but I did not see any red. I did not see the shape of the tail but it was black, or at least dark, otherwise I believe I would have noted its whiteness. I did not see the ventral area as the bird was flying directly away from me and angled upwards (Figure B1).

#### From Don Scheifler

It was a cool morning in Louisiana on October 27, 2019. I made my way through the bottomland forest, alternating between a slow walk and attentive pauses, hoping to hear the calls or double‐knocks of an Ivory‐billed Woodpecker. However, there was little sound of birds calling and almost no woodpeckers drumming. The quiet was occasionally broken by the distant boom of gunshots from deer hunters.

At mid‐day, while standing still and listening, I heard the rapid flaps of a bird approaching from ahead and to my right. The rate of the flaps was similar to those of a duck, but rather than the slurred flapping‐whirring sound of a duck, these wingbeats were very crisp. The sound of each flap was sharp and distinct from those before. Looking up, I saw a large bird flying perhaps 15 m (50 ft) above the ground, below the forest canopy but above the shorter trees. It had a hefty main body with long wings. The body of the bird was black, as well as the leading section of each wing. Most striking was the unmistakable bright white on the trailing portion of each wing, visible throughout each blurry flap. I was transfixed watching the bird fly past, focused on the wings. Because I had no habit of quickly snapping photographs, I never thought about lifting the camera hanging around my neck. I just stared as the bird disappeared, flying fast, straight, and level into the forest beyond my sight.

I stood there in near disbelief, struggling to decide what to do. Should I try to follow the bird? Should I sit down, immediately write a description of what I saw? Or, should I call someone? Who? I started texting my brothers about what I had seen. A minute or two later, I heard the same wingbeats coming back, then overhead. I only got eyes on the bird again when it was just past me. Again, a big black and white bird, with bright white on the back portion of each wing. It continued past me quickly and moments later swooped down a bit, then back up, spread its wings to slow, and landed in the fork of a tree maybe 38 m (125 ft) distant.

Glare from a bright, cloudy sky behind the tree made details hard to see, but I could make out the bird sitting in the tree fork. This time I raised my digital camera, aimed, pressed the photo button… and the auto‐focus refused to settle on the bird! Instead, the focus constantly shifted in and out due to intervening branches from other trees. I crouched down a little, tried again, and got the same frustrating result. I then reached into my pocket, pulled out my iPhone 6s, selected the camera icon, aimed in the general direction of the bird, and tapped for a picture. I switched to video mode, aimed… and then saw that the tree fork was empty. The bird appeared to be gone. I waited a few moments, then crept to the right, maintaining distance while partially circling the tree and hoping to see the bird clinging to the bole of the tree or maybe flying away. But the bird had vanished.

Looking at my photograph later, I was able to find what appeared to be the body of a bird in the fork of the tree, with the head apparently obscured. What is visible appears to include a white patch on the bird's lower back—a major field mark for identifying Ivorybills. The photograph is poor and will not be considered conclusive by itself, but it confirms to me that the bird I saw in flight that day was an Ivory‐billed Woodpecker (Figures C1 and D1).

#### From Peggy L. Shrum

From my field notes of February 8, 2020, I described the following:

On the trail at 0615 to (an undisclosed area) with Erik Hendrickson. We were attempting to reach and replace all of the ARUs (acoustic recording units) in the area, but as always, this area's terrain was rough and we were moving slowly. By around 0900 we had only reached the third ARU. We were on our way to the fourth, passing through large areas of standing water, with lots of backtracking and re‐orienting ourselves. As we slowly proceeded to the fourth point, I saw a large bird on a fallen log. I stopped; Erik was beside me to my right.

I whispered, “What is that?”

The bird was roughly 20–25 m ahead of us, and oriented facing our right. The head was obscured by vegetation, and the body was visible from the chest and back. My first impression was that I was seeing a black and white chicken, with black towards the front of body, and strikingly white plumage on the rear portion. No tail was visible.

As it began to sink in what I was looking at, I whispered to Erik, “I see the white.”

I then stepped to my left to use a large oak for cover as I also fumbled for my binoculars. Erik stepped to his right behind another tree. The bird then flew a few feet back, clung to the trunk of a tree, and perched vertically, 5–6 ft off the ground in bright sunlight. I very clearly saw what I can best describe as a *brilliantly white, “blunted heart”* shape. That is, if you drew a valentine heart and blunted the top and bottom such that the two top curves as well as the bottom point were flattened; that is what I saw on this perched bird's folded wings.

I did not make out the head or tail. The bird cocked to its left with a jerky motion and re‐oriented itself at an angle. It then flew and was gone instantly. I did not see the bird in flight, but I did see a burst of white as the bird‐initiated flight, and I was able to make out the shape of the bird's left wing before it disappeared. The wing was long and pointed in shape, more like a high aspect ratio wing shape. It was not at all rounded (Figure E1).

We waited still and quiet for about 20 min but heard or saw nothing more. I immediately made field notes and sketches, and we measured off the distances and the diameter of the tree. We took a lot of photographs, and placed several trail cameras, as well as an ARU at the location. The cameras did not produce any images, and the ARU did not record any putative Ivorybill sounds.

#### From Erik Hendrickson

My sighting of an Ivory‐billed Woodpecker occurred in mature bottomland hardwood forest on February 15, 2020, at about 0945. This was a horrible sighting in the sense that my description is lacking detail that I know others will want, but it was also one of the most incredible sightings in all my birding experiences, and the place, circumstances, what I saw, and behavior leaves no doubt in my mind as to identification. The following account is based on notes that I made in the field, except where otherwise indicated.

My search partner, Peggy Shrum, and I had walked in that day about 1.8 km (1.1 mile, although we probably covered at least 2.4 km (1.5 miles) or more skirting channels and other obstacles. Our purpose was to install a trail camera at a spot where Peggy had observed an Ivory‐billed Woodpecker one week prior, on February 8, 2020. One last channel blocked our route. On a day with lower water levels, we likely would have found a path forward at a shallow spot, or over an exposed log, but on this day, we went up and down the channel, not finding a good place to cross. Finally, Peggy decided to crawl across a long, narrow log and into a tangle of vines on the opposite side. The vines were so thick I was forced to follow on hands and knees also.

Many of the details in my account are not important in terms of what I observed, but they are the details that one remembers when something extraordinary happens: I reached the end of the log and stepped awkwardly and slowly through the vines, moving towards the clearing that was our destination. When finally clear of the vines, I bent forward to wipe the palms of my hands on my thighs, then straightened up and took hold of my smart phone that dangled from my neck. I looked down at the GPS app running on my phone and verified I was “on line” with our destination and raised my head.

Looking ahead for a landmark, I looked across a clearing, and saw a blur of wings. The blur was at ground level, or just above the ground. The blur was mostly white—part of it was brilliant white, and other parts (smaller parts) faded to grayish white. The blur seemed to be overall spherical. It was obviously a bird, and the blur went powerfully upwards (I estimated at an 80° angle) into the leafless crown of a tree.

I saw no field marks that we associate with Ivory‐billed Woodpecker: I did not see the head, or bill, or neck or body, or the tail—it was just a powerful, spherical blur of white wings, launching powerfully in a near vertical ascent. It was startling to see, and I suspect I startled the bird. It seemed to me an amazing display of power. It was larger and more powerful than any passerine, or any other bird I saw in the bottomland. It flew upward unlike any bird I have ever seen anywhere. It happened in a startling second.

None of my observations are considered “field marks.” However, they identified the bird. I whisper‐called for Peggy, and we stood behind a 1.2 m (4‐ft) diameter oak. In the tree canopy ahead, I saw a large, “dark and light” bird fly from right to left, but I did not see where it flew from, or where it flew to, and I did not see any details of the bird. I saw the bird “move” through the canopy again in a shorter flight, and it was again completely obscured before and after the movement.

Peggy and I finally decided to move forward, she to the left and me to the right of the big oak. Within a minute or two, Peggy saw “movement”—enough to tell the bird had launched, and called out, “It's gone.”

I had participated in searches in this bottomland hardwood forest for a cumulative 12 weeks, specifically looking for birds, and seeing all of the expected species. Although it is always difficult to judge size and distance in the field, several hours after my observation I noted that the bird I saw with an unobstructed view was the size of (or slightly smaller than) a Red‐shouldered Hawk, a very common species in our study area. I later determined that this puts my observation in the size range of American Crow, a species often used for size comparisons with Ivory‐billed Woodpecker.

The visual impact of large wings, beating rapidly to launch a large bird and sustain its escape flight, created the motion my eye and mind interpreted as blur. When I first saw the bird, it appeared to be on the ground; flew powerfully upward for a height I have estimated as 24 m (80 ft) and made short flights through the tree canopy—behavior inconsistent with herons, ducks, raptors, and kingfishers. In the two short canopy flights I observed, the bird was large enough to be detected, but it never perched, or paused, or lingered to engage in any activity. The quality of the bird's white coloration (mostly white, and its brightness) was significant; it was the dominant color that I saw, and comparable only to white egrets I have observed rarely in the bottomland. The bird wasn't bold, nor vocal; it wasn't mostly dark, or mostly blue like the common corvids in the area.

I understand that my sighting is awful, in so far as I saw none of what we consider classic field marks of an Ivorybill, and I had no opportunity to observe the bird for any length of time. However, I am also confident in identifying this bird as an Ivory‐billed Woodpecker.

#### From Jay Tischendorf, DVM

On Sunday, May 16, 2021, while engaged in fieldwork with the National Aviary and Project Principalis, I was alone and driving at approximately 32–40 km/h (20–25 mph) along a road within the bottomland hardwood swamp forest of Louisiana. At the time and location of this observation, the road was straight and there were no other vehicles in sight. At 1140, a black and white bird flew directly across the road in front of me. It had emerged from the mostly upland forest immediately adjacent to the roadway. The bird was roughly the size of a crow and was traveling east to west. It was flying steadily and level at approximately 6–9 m (20–30 ft) altitude. When I first saw it, the bird was approximately 6–9 m (20–30 ft) in front of the vehicle.

Almost instantaneously, upon seeing a black and white bird of this size, I knew it was either a Pileated or an Ivory‐billed woodpecker. My instantaneous and instinctive assumption was that this would be a Pileated Woodpecker, which are quite common in the area. Almost simultaneously, though, I realized that this bird seemed to have too much white and be too large to be a Pileated. I realized then I needed to focus and be very careful with this observation, taking in as much as I possibly could as fast and accurately as possible, for I realized this was quite possibly an Ivory‐billed Woodpecker and that this would likely be a fleeting experience. At the same time all of this was racing through my head, I was watching the bird, slowing the vehicle, and bringing it to a stop. The bird continued flying steadily into and onward through the upland forest on the far side of the road.

Overall, I observed this bird for 5–8 s as it flew approximately 45–68 m (50–75 yards) before disappearing into the forest. At one point, approximately 3–4 s into the observation, I literally stated aloud to myself, “Leading and trailing edge.” This was in reference to the pattern of white I was able to glimpse on the underside of the bird's wings as it passed overhead in front of me and then, as it continued moving, from an oblique perspective. The wings were not rounded and seemed too long to be a Pileated, and in fact even longer than those of a crow.

As noted above, the flight was steady, straight, and level. At no point was there any undulation in the aspect of flight, or interruption in the bird's steady and deliberate wingbeats. I can best describe the quality of the flight as “powerful and purposeful.” It was moving rapidly or perhaps even hastily, but, if I anthropomorphize, it did not seem to be in a panic. The bird never stopped flapping or erred from its straight and steady flight, even as it passed from the clear airspace of the roadway into and onward through the forest. Other than a slight twist or tilt to avoid a tree trunk, there was no pause to the wingbeats, no undulation to the flight path, and no fluttering or faltering quality to the flight. Additionally, the bird had a stiff wing movement, which I would describe more as a steady “pump” rather than a floppy, sloppy flap.

Upon losing sight of the bird, I jotted down notes about the observation and marked the location with my cellphone. For future reference, I also marked a nearby tree. I also launched a camera drone in hopes of possibly capturing the bird on film but did not encounter the bird again.

Habitat is the one and only factor associated with this observation that would suggest Pileated to me. This area is essentially all upland habitat, at least from what is visible from the road. However, the map of that area shows waterways or tributaries located in bottomland hardwood swamps within 1.6 km (1 mile) on either side of the road. Additionally, the drone footage I captured immediately after the observation actually shows a narrow strip of deciduous trees running through the upland forest at a 90° angle to the road and extending from the uplands at the road to bottomlands and hardwood swamp forest nearby.

Conveniently, within a few minutes of continuing my drive after the drone flight over the area, I saw 2–3 crows flying under similar conditions (altitude, habitat, sky conditions) to that of the mystery bird I had just seen. They provided solid reinforcement for comparing not just size but shape.

Finally, I would like to comment on the element of surprise when this sighting occurred. In this regard, one needs to understand that in ~5 year of involvement with Project Principalis, I have never entered these woods thinking I will even see an Ivory‐billed Woodpecker; I have always operated with the matter‐of‐fact belief I would actually *never* see an Ivorybill. In short, getting a glimpse of the Ghost Bird has never been my goal in working with Project Principalis, but rather just to know that I am helping in the search to keep its legacy alive. As would be true in any instance when some sort of wildlife suddenly manifests itself in front of you, I was entirely taken by surprise when I first saw this bird. The instantaneous and multi‐layered thoughts, reactions, and reflexes that occur when startled in this fashion are complex, particularly when it potentially involves a species considered extinct for the past eight decades.

I have concluded that this bird could only have been a Pileated or Ivory‐billed Woodpecker. It is only with extreme, and in the end truly unconvincing effort, that I can even come close to making it out to be the former: I am convinced I saw an Ivory‐billed Woodpecker.

#### From Mark Michaels, 2021

From my field notes, October 20, 2021: 0754 stakeout at tree number one, Ivory‐billed Woodpecker sighting, bird flying at canopy height east to west. Silhouette only, long neck and tail projections, rapid flight, and one clear wing tuck noted.

Before recording the above, I had yelled, “Ivorybill!” Not, “What was that?”, “Did you see that?”, or even “I think I saw one.” It was an expression of shock and certainty.

The sighting lasted ~3 s. Skies were overcast, and no field marks were noted on a couple of Pileateds that had flown by previously. The bird I saw did not remotely resemble a Pileated Woodpecker in profile, flight style, or speed.

My first impression when the bird entered my field of view was that it was a duck. Seeing the distinct wing tuck is what led to the shout. Wing tucks result from flap bounding, a flight style that is universal (or nearly so) in woodpeckers, including the Ivorybill.

In the aftermath of the sighting, I thought about what kind of duck it most closely resembled, and I came up with Common or Red‐breasted merganser as the best analogy. It is possible I subliminally noted a crest, but I do not have a conscious awareness of that. I looked at a field guide and thought, merganser was a good analogy, but the tail was too short.

I had always intellectually understood Tanner's reference to the Northern Pintail (*Anas acuta*) in comparison with Ivorybills. It is apt in terms of neck and tail projections, but less so in terms of body shape. This sighting deepened that understanding. Overnight, it struck me that the similarity in body structure to a diving duck might relate better to some of the swooping and diving we see in one of our drone videos of a purported Ivorybill.

I'm really adept at questioning myself, but this was not a mistake about the position of the field marks. The immediate default to duck, followed by the shock of seeing the wing tuck, would seem to rule out some kind of expectation bias.

I have had nagging doubts about several earlier possible sightings I have had of Ivorybills. I have spent countless hours in the field over 15 years without seeing anything I could be absolutely sure was an Ivorybill. My expectation of having an encounter, visual or auditory, on any given day is extremely low. Any possible encounter is exciting, but this one was a shock albeit a delightful one. If I were serious about keeping a life list of birds seen, this observation would be on it. That is a first for me.

## APPENDIX 2

### Audio recordings

- An extract from the last 6 min of recordings made by M. Courtman with P. Vanbergen at our Louisiana study area in March 2017 using a Zoom H4N handheld recorder. Because some calls seem to be closer than others are, we believe that two or more presumed Ivory‐billed Woodpeckers are present. Audiospectrograms appearing in Figure 1 are derived from calls at 8–9 s (Figure 1n) and the final 3 calls of the clip (Figure o, p, q) (Audio 2a).

b A putative Ivory‐billed Woodpecker double‐knock recorded in our study area by an AudioMoth acoustic recording unit on February 18, 2019 (with a Pileated Woodpecker calling in the distance). An audiospectrogram of this double‐knock is depicted in Figure 2a and a waveform in Figure 3a (Audio 2b).

c An anthropogenic double‐knock followed by an apparent distant response, consistent with an Ivory‐billed Woodpecker. Recorded by M. Courtman on March 15, 2017, using a Zoom H4N handheld recorder (Audio 2c).

d A double‐knock in apparent response to a calling Barred Owl (*Strix varia*) recorded on AudioMoth acoustic recording unit ~ February 23, 2019 (Audio 2d).

## APPENDIX 3

### 3.1

“Video” clip composed of photographs from a PlotWatcher Pro Game Surveillance System camera taken at 5‐s intervals showing three large woodpeckers foraging together on November 30, 2019. Unlike adult Pileated Woodpeckers that are territorial year‐round and typically drive young away from the territory as early as September, Ivory‐billed Woodpeckers reportedly show no indication of being strongly territorial, and offspring have remained with family groups for a full two years after fledging. Figure 4 (top), showing a bird with a pronounced white saddle, is extracted from this “video” clip, but most frames are ambiguous as to field marks (Video 3).

## APPENDIX 4

### 4.1

Time lapse “video” clip composed of photographs from a StealthCam taken at 30‐s intervals showing two large woodpeckers foraging together on October 12, 2021. The images are in silhouette, and field marks are not visible until one of the birds is high on the tree, at which point an apparent white saddle on the back can be seen; a white saddle can be seen simultaneously on the second bird clinging to the lower bole. As in Appendix 3, these large, crested woodpeckers demonstrate very active foraging movements on a branch at lower left before moving up the tree. Foraging is acrobatic; the birds hang from vines and cling to the tops, sides, and undersides of the branches (Video 4).

## APPENDIX 5

### 5.1

A half‐speed video filmed by Autel Evo 2 6K drone on February 23, 2021 shows a large woodpecker fly in from the extreme lower left of the screen, land on a tree trunk at center screen, and then make its way along a horizontal branch stub before taking off from the end of the branch. The putative Ivory‐billed Woodpecker displays a well‐defined white saddle while moving across the branch, and in flight the dorsal wing surfaces show extensive white divided by a prominent black body, and a black leading edge to the wing. Calculations appearing in Appendix 8 suggest that this bird has a wingspan of 74.7 ± 7.9 cm (29.4 in ±3 in) (Video 5).

## APPENDIX 6

### 6.1

In a subsequent flight by the same bird appearing in Appendix 5, here shown at full speed, the presumed Ivory‐billed Woodpecker flies across the lower foreground while flight bounding, including a remarkable upward bound, then spreads its wings as it prepares to land on an upright tree trunk allowing a full dorsal view (Video 6).

## APPENDIX 7

### 7.1

A cropped view at half‐speed of the landing seen in Appendix 6. Readily visible features include extensive white on the dorsal surface of the wings, and a clear black body dividing the wings in all frames where it is visible. Upon landing, the putative Ivory‐billed Woodpecker shows a clear white saddle across the lower back, even as it moves across the branch, disappears momentarily around the back side of the branch, and then reappears. Calculations appearing in Appendix 8 suggest that this bird has a wingspan of 74.7 ± 7.9 cm (29.4 ± 3 in) (Video 7).

## APPENDIX 8

### 8.1

Calculating wingspan of a putative Ivory‐billed Woodpecker from a drone video shot on February 23, 2021 in Louisiana

1. Using a mm ruler on a computer monitor (Dell SE2419H), we made three measurements of the bird's wingspan (WS) in four frames where the wings were judged to be fully extended (*n* = 12 measurements).
  The measured frames were 8, 9, 10, and 11 frames prior to landing. In these four frames, the wingspan appeared maximum and uniform.

**Table:**

	1st	2nd	3rd	Mean
Frame 8 prior to landing	14.1	14.2	14.0	14.1
Frame 9 prior to landing	14.8	14.9	15.0	14.9
Frame 10 prior to landing	15.0	15.0	14.9	15.0
Frame 11 prior to landing	14.0	14.1	14.0	14.0

Data from these measurements suggested that in frames 8 and 11 the wings are somewhat less fully spread, so these measurements were eliminated, and we calculated the mean of the six measurements from frames 9 and 10:

Wingspan measurement = 14.9 mm

Estimate of error of measurement = 0.5 mm

Wingspan measurement = 14.9 ± 0.5 mm (±3.3%)

2 Using a mm ruler on the same monitor with the same frame and same zoom factor, we made three measurements of the DBH (diameter at breast height) of the tree. These measurements were: 14.5, 13.2, and 13.4 mm. We took the mean of these measurements.
  DBH measurement = 13.7 mm
  Estimate of error of measurement error = 1 mm
  DBH measurement = 13.7 ± 1 mm (±7.3%)
3 Note that all measurements are for the random zoom factor used on the computer monitor in this particular instance; if repeated on this or any other computer monitor, the zoom factor will surely be different. What is important is the ratio of the wingspan to the DBH, not the absolute value of the measurements themselves.
  The ratio of WS to DBH = 14.9/13.7 = 1.09 ± 0.1 (±10.6%)
4 Since actual DBH measured at the tree = 74 cm, our best preliminary estimate of WS (before taking into account the difference in distance from drone to bird, and drone to DBH point) is:
  WS = (ratio of WS to DBH) (actual DBH)
  = (1.09 ± 0.1) (74 cm)
  = 80.7 cm ± 8.5 cm
  = 31.8 in ± 3 in
5 However, the bird was slightly closer to the drone than the point on the trunk where the DBH was measured. To take into account this difference, we compared the distances from the drone to the bird, and the drone to the DBH point:
  To calculate the distance from the drone to the bird, we used the Pythagorean theorem, c^2^ = a^2^ + b^2^.
  We knew the height of the drone was 350 ft (106.7 m), and the horizontal (along the ground) distance from the drone to the tree (and to the bird) was measured by a subsequent drone flight as 450 ft (137.2 m). We estimated that the bird was 80 ft (24.4 m) above ground based on prior estimates of tree heights in our search area.
  So: Distance, *x*, from drone to bird is:
  *x* = sqrt [(350 − 80 ft)**2 + (450 ft)**2)] = 525 ft (160.0 m)
6 Similarly, we calculated the distance from the drone to the DBH point.
  We knew the height of the drone was 350 ft (106.7 m), and we estimated the horizontal distance from the drone to the tree (where DBH was measured at 130 cm = 4.25 ft above ground) was 450 ft (137.2 m) (as measured by a subsequent drone flight).
  So: Distance, *y*, from drone to DBH is:
  *y* = sqrt [(350 − 4.25 ft)**2 + (450 ft)**2)] = 567 ft (172.8 m)
7 Because the bird is closer to the drone camera than the DBH point is, the bird appears to be larger than it actually is by a factor of 567/525 = 1.08
8 So, the actual wingspan (WS) of the bird is:
  WS = 80.7 cm ± 10.6%/1.08
  = 74.7 cm ± 7.9 cm
  = 29.4 in ± 3 in
9 Assumptions in our calculation include:

- The bird with outstretched wings is square to the camera (i.e., perpendicular to the line of sight from the camera);
- Trunk diameter measured on September 30, 2022, is the same as on the date of the drone video filmed on February 23, 2021;
- The trunk cross section is a perfect circle;
- White blur and black wing tips that may or may not be visible in the video frames, do not affect the measurements in a significant way;
- The horizontal projected distance from the drone to the bird, and from the drone to the DBH point, is 450 ft (137.2 m); the height of the bird in frames 9 and 10 is 80 ft (24.4 m). The estimate of wingspan is sensitive to the relative measurements on a computer monitor of the bird's wingspan and the tree's DBH, but is insensitive to the distance from the drone to the bird and the tree;
- The bird is 80 ft up in frames 9 and 10;
- The horizontal projected distance from drone to DBH point and to the bird are the same, where the “horizontal projected distance” is the part of the “distance” represented ONLY by the horizontal part. We calculated above the “actual” distance that lies along the hypotenuse of a “sloped” line of sight.

## APPENDIX 9

### 9.1

A full speed video shot on October 20, 2022 with an Autel Evo II drone with a 6K camera. At ~6 s a Red‐headed Woodpecker enters the middle foreground, briefly lands, and then flies off at ~12 s in the direction from which it arrived. While briefly perched, one can even see its tiny saddle on the trunk. The Red‐headed Woodpecker is identified by its small size, and the dorsal wing surfaces of the wings show a black leading edge to the wing with extensive white, but the white is continuous from wing‐to‐wing because of the presence of the prominent white rump. Beginning at 32 s two putative Ivory‐billed Woodpeckers enter the frame from the mid‐right margin. These two birds, clearly interacting, display field marks consistent with Ivory‐billed Woodpeckers, including the dorsal wing surfaces of the putative Ivorybill with extensive white divided by a prominent black body, and a black leading edge to the wing (Video 9).

## APPENDIX 10

### 10.1

Drone video clip filmed at our Louisiana study site depicting the landing of a Pileated Woodpecker on an upright snag. Here, the Pileated Woodpecker displays a very small amount of white on the dorsal surface of the wings compared to the putative Ivory‐billed Woodpecker in Appendices 6 and 7, or the Red‐headed Woodpecker in Appendix 12. An image extracted from this drone video clip also appears in Figure 10 (Video 10).

## APPENDIX 11

### 11.1

A zoomed and cropped, three‐quarters speed video shot of the two putative Ivory‐billed Woodpeckers interacting in Appendix 9 (Video 11).

## APPENDIX 12

### 12.1

Drone video clip filmed at our Louisiana study site depicting the landing of a Red‐headed Woodpecker on an upright snag. Here, the Red‐headed Woodpecker shows a black leading edge to the wing with extensive white, but the white is continuous from wing‐to‐wing because of the presence of the prominent white rump. Compare with the similar landing sequence of a putative Ivory‐billed Woodpecker in Appendices 6 and 7, where the bird shows extensive white divided by a prominent black body, and a black leading edge to the wing. An image extracted from this drone video clip also appears in Figure 10 (Video 12).
